# MicroRNA-199b-5p Impairs Cancer Stem Cells through Negative Regulation of HES1 in Medulloblastoma

**DOI:** 10.1371/journal.pone.0004998

**Published:** 2009-03-24

**Authors:** Livia Garzia, Immacolata Andolfo, Emilio Cusanelli, Natascia Marino, Giuseppe Petrosino, Daniela De Martino, Veronica Esposito, Aldo Galeone, Luigi Navas, Silvia Esposito, Sara Gargiulo, Sarah Fattet, Vittoria Donofrio, Giuseppe Cinalli, Arturo Brunetti, Luigi Del Vecchio, Paul A. Northcott, Olivier Delattre, Michael D. Taylor, Achille Iolascon, Massimo Zollo

**Affiliations:** 1 CEINGE, Centro di Ingegneria Genetica e Biotecnologia Avanzate, Naples, Italy; 2 Dipartimento di Chimica delle Sostanze Naturali, Università di Napoli “Federico II”, Naples, Italy; 3 Dip. di Scienze Cliniche Veterinarie - Sez. di Clinica Chirurgica, Università di Napoli “Federico II”, Naples, Italy; 4 Diagnostic Imaging Department, University of Naples “Federico II” and IBB-CNR, Naples, Italy; 5 Unité de Génétique et Biologie des Cancers, Institut Curie, Paris, France; 6 Anatomia Patologica Ospedale Pausilipon AORN Santobono-Pausilipon, Naples, Italy; 7 Pediatric Neurosurgery, Santobono-Pausilipon Children's Hospital, Naples, Italy; 8 Department of Biomorphological and Functional Sciences, Institute of Biostructures and Bioimages of the National Research Council, University Federico II, Naples, Italy; 9 DBBM, Dipartimento di Biochimica e Biotecnologie Mediche, Università di Napoli “Federico II”, Naples, Italy; 10 Arthur and Sonia Labatt Brain Tumor Research Centre, The Hospital for Sick Children, Ontario, Canada; 11 Facoltà di Scienze Biotecnologiche, Università di Napoli “Federico II”, Naples, Italy; Ordway Research Institute, United States of America

## Abstract

**Background:**

Through negative regulation of gene expression, microRNAs (miRNAs) can function in cancers as oncosuppressors, and they can show altered expression in various tumor types. Here we have investigated medulloblastoma tumors (MBs), which arise from an early impairment of developmental processes in the cerebellum, where Notch signaling is involved in many cell-fate-determining stages. MBs occur bimodally, with the peak incidence seen between 3–4 years and 8–9 years of age, although it can also occur in adults. Notch regulates a subset of the MB cells that have stem-cell-like properties and can promote tumor growth. On the basis of this evidence, we hypothesized that miRNAs targeting the Notch pathway can regulated these phenomena, and can be used in anti-cancer therapies.

**Methodology/Principal Findings:**

In a screening of MB cell lines, the miRNA miR-199b-5p was seen to be a regulator of the Notch pathway through its targeting of the transcription factor HES1. Down-regulation of HES1 expression by miR-199b-5p negatively regulates the proliferation rate and anchorage-independent growth of MB cells. MiR-199b-5p over-expression blocks expression of several cancer stem-cell genes, impairs the engrafting potential of MB cells in the cerebellum of athymic/nude mice, and of particular interest, decreases the MB stem-cell-like (CD133+) subpopulation of cells. In our analysis of 61 patients with MB, the expression of miR-199b-5p in the non-metastatic cases was significantly higher than in the metastatic cases (P = 0.001). Correlation with survival for these patients with high levels of miR-199b expression showed a positive trend to better overall survival than for the low-expressing patients. These data showing the down-regulation of miR-199b-5p in metastatic MBs suggest a potential silencing mechanism through epigenetic or genetic alterations. Upon induction of de-methylation using 5-aza-deoxycytidine, lower miR-199b-5p expression was seen in a panel of MB cell lines, supported an epigenetic mechanism of regulation. Furthermore, two cell lines (Med8a and UW228) showed significant up-regulation of miR-199b-5p upon treatment. Infection with MB cells in an induced xenograft model in the mouse cerebellum and the use of an adenovirus carrying miR-199b-5p indicate a clinical benefit through this negative influence of miR-199b-5p on tumor growth and on the subset of MB stem-cell-like cells, providing further proof of concept.

**Conclusions/Significance:**

Despite advances in our understanding of the pathogenesis of MB, one-third of these patients remain incurable and current treatments can significantly damage long-term survivors. Here we show that miR-199b-5p expression correlates with metastasis spread, identifying a new molecular marker for a poor-risk class in patients with MB. We further show that in a xenograft model, MB tumor burden can be reduced, indicating the use of miR199b-5p as an adjuvant therapy after surgery, in combination with radiation and chemotherapy, for the improvement of anti-cancer MB therapies and patient quality of life. To date, this is the first report that expression of a miRNA can deplete the tumor stem cells, indicating an interesting therapeutic approach for the targeting of these cells in brain tumors.

## Introduction

MicroRNAs (miRNAs) are single-stranded RNAs of ∼22 nucleotides in length, and they constitute a novel class of gene regulators [1]. In animals, miRNAs have regulatory effects through their binding to imperfect complementary sites within the 3′-untranslated regions (3′UTRs) of their mRNA targets. Altered expression of many miRNAs is seen in several tumor types: e.g. B-cell lymphomas (clustered miR-17) [2], [3], malignant lymphomas (miR-15a, miR-16-1; targeting BCL2) [4], glioblastoma tumors (miR-21up-regulation) [5], colorectal neoplasia (miR-143, miR-145 down-regulated) [6], lung cancer (miR-29) [7], and breast cancer (miR-10b) [8], with several more tumor types under analysis.

Here, we have focused on the most common of malignant brain tumors, medulloblastomas (MBs). MBs appear to originate from stem cells and from granule neuron precursors in the external granule layer of the cerebellum [9], or alternatively, from multipotent precursors in the ventricular zone of the cerebellum [10]–[12]. From a clinical point of view, current multimodal treatments include radical surgical resection followed by radiation and chemotherapy. While these treatments can improve the survival rate, MB remains incurable in about one third of these patients. The main cause of death is recurrence associated with tumor dissemination, at which point current therapeutic options have little efficacy [13]. These treatments are also toxic and can lead to long-term disabilities [14]–[16]. Consequently, there is a substantial need for novel, effective, low-toxicity therapies for children with medulloblastoma.

MB cells can also contain functionally important subsets of cells with stem-like properties that are uniquely able to propagate tumor growth [17], [18]. Recent studies have demonstrated that both progenitors and stem cells can respond to the Sonic-Hedgehog (Shh) pathway and can serve as cells of origin for MBs [19].

Notch activity has been shown to regulate granule-cell progenitors from which MBs arise, with Notch2 gene copy numbers increased in 15% of MBs [12], [20]. Persistent expression of *bHLH HES1*, the principal Notch-responsive gene, prevents both migration of neural progenitor cells out of the ventricular zone and expression of neuronal markers [21]. Mice lacking *Hes1* show premature neurogenesis, seen as up-regulation of neural bHLH transcription factors, which results in major neural-tube defects [22]; progenitor cells derived from these mice have impaired self-renewing potential, with a commitment towards a neuronal lineage [23].

Significantly, expression of HES1 in MB has been associated with worse clinical outcome [24]. Of note, an interplay with Shh in granular cell precursor development has been postulated [25], as has cross-talk with other pathways, e.g. the polycomb group gene *BMI-1* [26].

Our approach started with a search for miRNAs (miRNA registry) with target genes involved in the Notch pathway, and we identified miR199b-5p as targeting HES1, the Notch effector. We show that miR-199b-5p expression is lost in metastatic patients and postulate a mechanism of regulation following epigenetic silencing through methylation processes occurring during carcinogenesis, identifying a new molecular marker for a poor-risk class in patients with MB. MiR199b-5p expression specifically impairs the cancer-stem-cell (CD133+) population, which results *in vivo* in impairment of MB tumor development in the cerebellum xenograft mouse model, thus providing proof of concept. As microRNAs have been recently shown to be useful tools to silence cancers, they might be able to fill this gap through their control of multiple target genes. We provide here evidence for the use of miRNAs in the clinic, with our anti-tumour therapy targeting of cancer stem cells by miR-199b-5p.

## Results

### Hsa-miR-199b-5p silences HES1 expression via 3′UTR binding

Our *in-silico* analysis of the mirBase targets database [27] was directed towards identification of miRNAs potentially targeting HES1, an effector of the Notch pathway, a fundamental mechanism in the regulation of MB cell proliferation. MiR-199b-5p and miR-199a-5p were the better scoring miRNAs, and were predicted to bind the 3′UTR of human bHLH HES1. We focused on miR-199b-5p due to its ability to decrease HES1 expression under transient over-expression, as compared to miR-199a-5p (Text S1, Fig. S1A, B). MiR-199b-5p is lost in lung tumors [28] and it has been mapped to a genomic region deleted in bladder cancer [29]. MiR-199a* (also known as miR-199a-5p, and with an identical sequence to miR-199b-5p) is also reduced in hepatocellular carcinoma [30]–[32]. The analysis of miR-199b-5p expression in two MB cell lines, human adult tissue and mouse cerebellum, is shown in Text S1, Fig. S1C, D and S2A, B.

To determine whether HES1 is a target of miR-199b-5p, the HES1 3′UTR was cloned downstream of a luciferase reporter gene vector; pre-miR-199b-5p was also cloned in a mammalian expression vector (see Text S1). HEK-293 cells were then transfected with the relative luciferase activity showing that miR-199b-5p co-transfection decreased reporter gene activity, thus indicating binding with the 3′UTR and destabilisation of productive translation of luciferase mRNA (Text S1, Fig. S1E). As controls, HES1 3′UTR mutated in the miR-199b-5p binding site was not affected by miR-199b-5p (Text S1, Fig. S1E), and when a 2-O′-methyl oligoribonucleotide (2-OM) complementary to mature miR-199b-5p was co-transfected, it counteracted the effects of miR-199b-5p transfection, restoring reporter activity (Text S1, Fig. S1E). Similar results were obtained in Daoy MB cells (Text S1, Fig. S1F).

To determine the role of miR-199b-5p in MB cell biology, the miR-199b-5p expression construct was transfected into Daoy cells, and several stable clones over-expressing miR-199b-5p were selected. Over-expression was confirmed by real-time PCR (Text S1, Fig. S1G). Three clones demonstrated reduced HES1 protein levels, one of which (199bSC1) showed no detectable HES1. The 199bSC1 and 199bMC1 clones were selected for further investigation (Text S1, Fig. S1H). These effects of miR-199b-5p on HES1 protein expression were not restricted to the stable clones or Daoy cells, as D283MED cells transiently transfected with the expression construct for miR-199b-5p also showed reduced HES1 levels (Text S1, Fig. S1B).

To strengthen these findings, the 199bSC1 clone was transfected with 2-OM antisense to miR-199b-5p and used as a negative control (Text S1, Fig. S1I). Here, HES1 levels were restored, suggesting 2-OM block of HES1 repression by miR-199b-5p, providing further confirmation that miR-199b-5p targets HES1 directly. Other potential targets of miR-199b-5p were also investigated in this way (Text S1, Fig. S2C, D).

### Over-expression of miR-199b-5p reduces cell proliferation and impairs the clonogenic potential of MB cell lines

**Figure 1:**
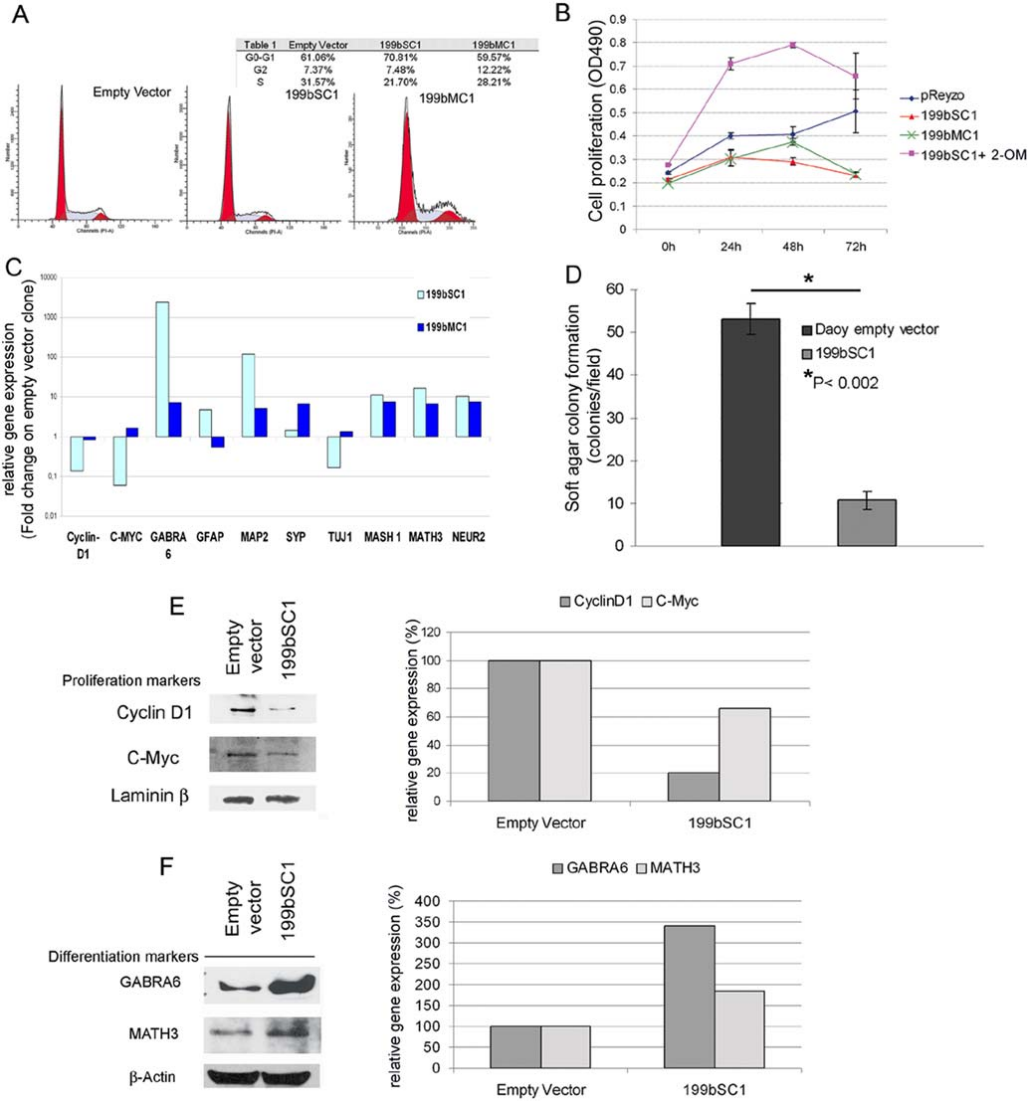
*In-vitro* function of miR199b-5b on proliferation and differentiation of Daoy MB cells. A) Representative FACS analysis with propidium iodine showing a decrease in the percentages of cells in S phase and an increase in G0-G1 for the stable 199bSC1 clone. The absence of shoulder signals with the G0-G1 red peak in the 199bSC1 and 199bMC1 clones excludes apoptotic processes. B) Proliferation assays by 3-4,5-dimethylthiazol-2-yl-5-3-carboxymethoxyphenyl-2-4-sulfophenyl-2H-tetrazolium salt (MTS), showing decreased proliferation rates of the stable 199bSC1 clone (red triangles) and the 199bMC1 clone (green crosses), compared to the stable clone with empty vector alone (blue diamond). This effect of miR-199b-5p over-expression was reduced by 2-OM transfection (pink squares). The data shown are means±SD from two independent experiments, each carried out in triplicate. C) Rapresentative mRNA expression of differentiation (e.g. MASH1, MATH3 and NEUROGENIN 2) and proliferation (e.g. c-MYC, CYCLIN D1) markers upon miR-199b-5p over-expression, as revealed by real-time PCR, comparing the stable 199bSC1 and 199bMC1 clones with empty vector clone. D) The stable 199bSC1 clone for miR-199b-5p shows impaired colony formation in soft agar assays. The plot shows colonies counted as means±SD from three independent experiments, each carried out in triplicate. E) Representative Western blot using anti-c-Myc and anti-cyclin D1 antibodies show these two proteins to be down-regulated in the stable 199bSC1 clone; see also the densitometric analysis in the right panel. F) As for E, showing GABRA6 and MATH3 proteins up-regulated after miR-199b expression, suggesting induction of differentiation. Anti-Laminin-β antibodies were used to normalized nuclear c-Myc and cyclin D1 protein expression. Anti-β-Actin antibodies were used to normalized cytoplasmic GABRA6 and MATH3 proteins expression.

The 199bSC1 and 199bMC1 clones had reduced proliferation rates under standard culture conditions, when compared to the control clone. Thus, we looked for potential cell-cycle alterations in these clones. FACS analysis on the 199bSC1 clone showed a 31% decrease in S-phase fractions, and an increase in cells in G0-G1 of 15%, as compared to the empty vector clone (Fig. 1A). This suggested that exit from the cell cycle has a role in the reduced proliferation rate of the Daoy cell 199bSC1 clone. In contrast, the 199bMC1 clone did not show significant changes in its cell cycle. We believe these findings to be due to an overall lower efficiency of 199bMC1 for reducing HES1 protein levels. These cell-cycle phenotypes translated into decreased proliferation rates *in vitro*, as evaluated by *in-vitro* proliferation assays comparing the 199bSC1 and 199bMC1 clones with an empty-vector clone and a transient transfectant for 2-OM designed against miR-199b-5p (Fig. 1B). Both the 199bMC1 and the 1999SC1 clones showed markedly reduced proliferation rates. Transfection of this antisense 2-OM induced a marked increase in proliferation of the stable 199bSC1 clone, in agreement with restored expression levels of HES1. These results on Daoy cell proliferation were confirmed also with D283 and ONS76 cells (Text S1, Fig. S2F). The effects of miR-199b-specific 2-OM were also confirmed in wild-type Daoy cells, where it potentially acts on the endogenous miR-199b (Text S1, Fig. S2G).

The effects of the induction of miR-199b-5p were evaluated on molecular markers of proliferation and differentiation by a real-time approach. As illustrated in Fig. 1C, MAP2, which is mostly expressed in mature neurons [33], was up-regulated in the stable 199bSC1 and 199bMC1 clones. Similarly, Daoy cells have been reported to express GFAP after differentiation with phenylbutyrate [34], and in the stable 199bSC1 clone, GFAP levels were increased. Overall, the picture of gene expression in our stable cell line over-expressing miR-199b-5p is in agreement with the phenotype that has been seen in the brain of the *Hes1−/−* mouse [23]. Among the other genes, GABRA6, a marker of cerebellar granule cell differentiation, was also significantly over-expressed in the stable clones (Fig. 1C).

A fine-tuned cascade of positive and negative bHLH transcription factors is central to neurogenesis, with genes such as *MASH1*, *MATH3* and *NGN2* inducing neurogenesis, and HES1-mutant mice showing up-regulation of these activator-type bHLHs [35]. Both miR-199b-5p stable clones showed increases in expression of pro-neural bHLH. In agreement with their reduction in proliferation rate, the proliferation markers c-Myc and cyclin D1 were decreased. Of the two clones analysed, 199bSC1 showed the more consistent phenotype; we explain these results by the more efficient HES1 silencing in this clone.

Since the miR-199b-5p stable 199bSC1 clone showed a stronger and more consistent phenotype, we next examined it in a standard clonogenic assay, to determine whether anchorage-independent growth was affected by miR-199b-5p. Here, there was an 80% reduction in colony formation potential, compared to the empty-vector clone (Fig. 1D). In agreement with this reduction in proliferation rate, the proliferation markers c-Myc and cyclin D1 were decreased (Fig. 1E). We confirmed also at the protein level that GABRA6 and MATH3 were up-regulated in the 199bSC1 clone (Fig. 1F); the latter is known to be directly repressed by HES1 [35].

### MiR-199b-5p depletes the side population compartment in the Daoy cell line, and negatively regulates MB tumor stem-cell populations

The Notch pathway has been linked to the fraction of MB tumor cells that harbour precursor stem-cell markers [36], and HES1 has a role in self-renewing of multipotent progenitor cells [23]. This side population (SP) of tumor cells has a role in the engrafting of a tumor in animal models [37]. We thus examined the influence of miR-199b-5p on the population of tumor cells that exclude the Hoechst 33342 dye, a strategy to identify these SP cells. This was determined by flow cytometry in the Daoy cell line, the SP of which accounts for up to 4.9% of the cells, as compared with verapamil-treated cells (the negative control) (Text S1, Fig. S3A, B). This verapamil treatment was based on verapamil inhibition of the ion-pumps responsible for Hoechst 33342 dye exclusion, thus enabling the SP cells to be seen [38]. Staining of 199bSC1 and 199bMC1 cells indicated that the SP was ablated, as there were close to no differences between the Hoechst-treated sample and the Hoechst plus verapamil samples (Text S1, Fig. S3C–F).

**Figure 2:**
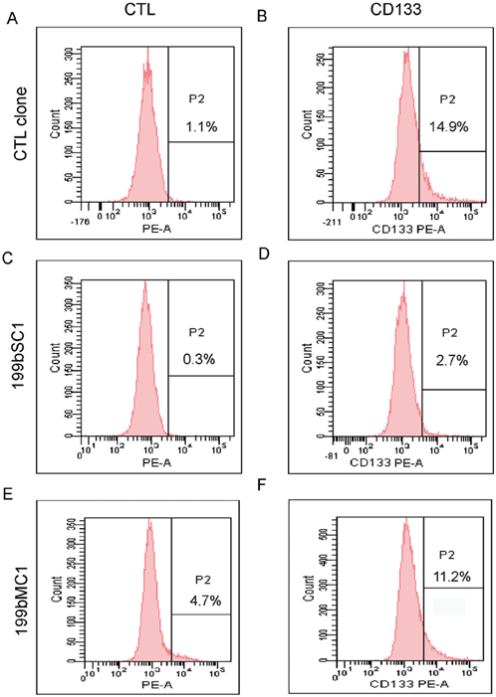
Over-expression of miR-199b-5p decreases the CD133+ compartment of Daoy cells. A–F) Representative FACS analyses of the stable 199bSC1 (D) and 199bMC1 (F) clones showed a decrease in the percentage of cells that were positive for the stem cell marker CD133 (B); A, C and E are negative controls (no antibody).

It is also known that central nervous system tumor stem cells express the CD133 antigen, and that these cells are uniquely capable of tumor formation in NOD-SCID mice [18], [39]. Additionally, the Notch pathway has a central role in the self-renewing process, with its inhibition leading to depletion of CD133-positive (CD133+) Daoy cells via induction of apoptosis of progenitor-like cells [36]. Recently it was shown that CD133+ Daoy cells promote tumor growth in the flank of nude mice, while CD133− cells do not [40]. For these reasons, we evaluated the CD133 positivity of Daoy cells as compared to the stable 199bSC1 and 199bMC1 clones (Fig. 2). Here, the wild-type cells were 14.8% CD133+, while in the stable 199bSC1 and 199bMC1 clones this was reduced to 2.4% and 6.5% CD133+, respectively (Fig. 2C–F). This thus demonstrated a role for miR-199b-5p in negative regulation of this fraction of tumor-initiating cells.

### HES1 over-expression rescues the miR-199b-5p clone phenotype

**Figure 3:**
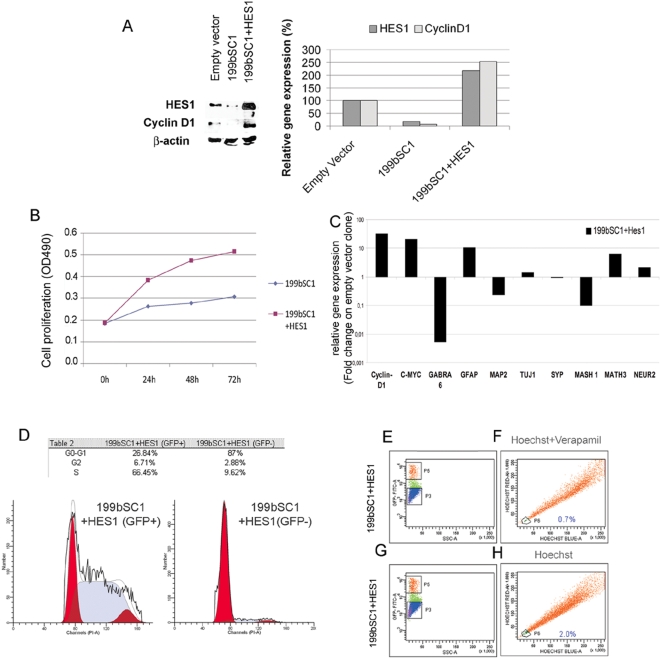
The effects of miR-199b-5p over-expression are reversed by HES1 transfection. A) Representative Western blot and densitometric analysis showing that the rescue of HES1 expression by over-expression of HES1 cDNA in the stable 199bSC1 clone restores cyclin D1 expression. B) Proliferation assay by 3-4,5-dimethylthiazol-2-yl-5-3-carboxymethoxyphenyl-2-4-sulfophenyl-2H-tetrazolium salt (MTS) showing increased proliferation rate of the stable 199bSC1 clone with HES1 expression (dark blue squares), compared to the stable 199bSC1 clone alone (light blue diamonds). The data shown are means±SD from two independent experiments, each carried out in triplicate. C) Rapresentative mRNA expression of differentiation (e.g. MASH1, MATH3 and NEUROGENIN 2) and proliferation (e.g. c-MYC, CYCLIN D1) markers upon cell-rescue with HES1 in the stable 199bSC1 clone, comparing with wild-type cells, as revealed by real-time PCR. The expression of GABRA 6 and MAP2 are down-regulated in the rescue experiment. D) Representative FACS analysis of the HES1-expressing stable 199bSC1 clone shows an increase in the fraction of cells in S phase, and a decrease in G1, with respect to the stable 199bSC1 clone alone. E–H) The effect of miR-199b-5p expression on the SP of Daoy cells is reversed by HES1 re-expression. 199bSC1 stable clone was transfected with HES1 cDNA and a GFP-coding plasmid, then treated after 48 hours with Hoechst and verapamil (E–F) or Hoechst alone (G–H) and analyzed by FACS. The GFP-positive cells (P5 gate), panel E and G, were analysed for the presence of dye-excluding-cells (F–H, P6 gate). The untransfected GFP negative cells (P3 gate), were not analised.

To confirm that the cellular phenotype is directly correlated with down-regulation of HES1, we performed *in vitro* cell-rescue experiments. We chose to rescue the more consistent phenotype, shown by the stable 199bSC1 clone. When full-length HES1 cDNA was cloned and transfected into the stable Daoy-cell 199bSC1 clone, HES1 expression was restored, as evaluated by immunoblotting (Fig. 3A). We also noted the re-expression of cyclin D1, which was down-regulated in the stable 199bSC1 clone. Restored HES1 expression also reversed the effects of miR-199b-5p on cell proliferation (Fig. 3B). At the same time, the transfection of HES1 cDNA into the 199bSC1 clone decreased induction of pro-neural bHLHs and differentiation markers, and increased levels of proliferation genes (Fig. 3C). Furthermore, the transient transfection of HES1 cDNA into the stable 199bSC1 clone led to an increase in the percentage of cells in S phase, and a removal of the block on the G0-G1 phase (Fig. 3D). This was opposite to the effects seen in the stable 199bSC1 clone over-expressing miR-199b-5p (see Fig. 1C and Fig. 3C). The effects of restored expression of HES1 on the SP of the stable 199bSC1 clone were also evaluated. When the HES1 transfected cells (GFP positive) were evaluated for their ability to exclude Hoechst dye, they showed a minimum increase in SP, of up to 1.3%. Although higher than that measured for the stable 199b clones (Fig. 3E–H), this was still below the level of SP cells for the wild-type cells (Text S1, Fig. S3). This is probably due to the short time of re-expression of HES1 after transient transfection, which might not be enough to allow for complete phenotype rescue. We then tried to rescue the effects on the CD133 compartment; however, transient transfection of HES1 in the 199bSC1 clone did not result in a significant increase in CD133+ cells. We believe that this is due to the short transient transfection time, which does not allow a re-organization of Daoy cell hierarchy.

### Tumor growth is reduced in xenografts derived from the stable 199SC1 clone

**Figure 4:**
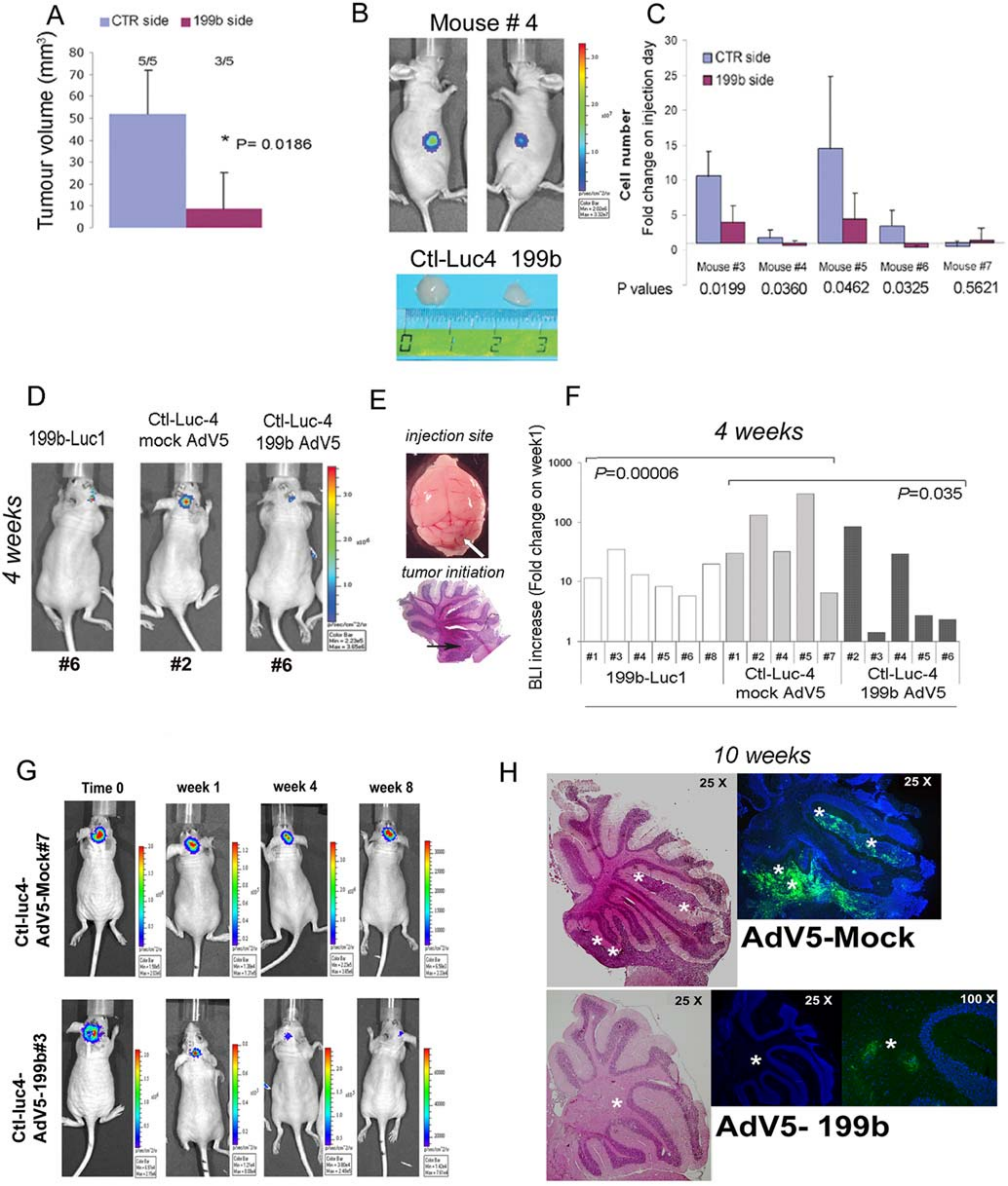
Decreased tumorigenicity of Daoy cells over-expressing miR-199b-5p. A) Xenograft experiment over nine weeks, following s.c. injection of five mice with control Daoy cells (CTR side) and Daoy cells over-expressing miR-199b-5p (199b side). With CTR cells, tumors were detectable macroscopically in 5/5 mice; with over-expressing cells, in 3/5 mice. Total tumor volume difference between the CTR and 199b injected sides after nine weeks of growth was statistically significant, as indicated (means±SD). B) Photon emission of mouse #4 after nine weeks of tumor growth. The 199b side (199bLuc-1) had a consistently lower photon emission than the control side (Ctl-Luc-4). The comparisons of the tumor dimensions are also shown. C) When photon emission (see B) was converted to cell number; one of five mice showed no significant differences (means±SD) (two tailed unpaired *t* test). D) Photon emission of 3 mice injected in the fourth ventricle with respectively: 199bLuc-1, Ctl-Luc-4 infected with mock AdV5, and Ctl-Luc-4 infected with a miR-199b-5p AdV5. E) Entire brain of animal and site of injection (white arrow). Hematoxylin/eosin staining of cerebellum; black arrow, site of initiation of tumorigenesis. F) BLI of injected mice as for D, after one month of tumor growth. The differences between the groups are statistically significant. G) BLI of two selected mice showing development of tumor burden with Ctl-luc4-AdV5-Mock#7, and reduction with Ctl-luc4-AdV5-199b#3 over time (0–8 weeks). H) Hematoxylin-eosin staining of cerebellum from AdV5-Mock#2 and AdV5-199b#3 animals at 10 weeks post orthotopic injection, showing tumors cells surrounding external granular layer and within the IV ventricle (white star). In blue, DAPY staining, together with GFP detection. Magnification as indicated.

The findings here indicated that miR-199b-5p is a potential inhibitor of tumor formation. Therefore, to investigate the role of miR-199b-5p in an *in vivo* tumor model, we stabilized the 199bSC1 and control Daoy clones with an expression vector carrying luciferase cDNA. The clones obtained were tested for luciferase expression levels and also validated for retention of the parental phenotype, in terms of both HES1 and miR-199b-5p expression (see Text S1, Fig. S4A–D). These stable 199b-Luc1 and Ctl-Luc-4 Daoy clones were then injected into the left and right flanks, respectively, of five athymic nude/nude mice. Tumor growth was evaluated by weekly *in-vivo* bioluminescence imaging (BLI) of injected mice. At eight weeks, all of the mice showed visible masses in each control flank, while only three flanks injected with 199b-Luc1 showed tumor engrafting. Overall, a significant difference in tumor volumes between control flanks and miR-199b-5p flanks was seen (Fig. 4A). The bioluminescence measurements showed significant reductions in emission for the miR-199b-5p sides during tumor growth, as compared with the control sides (e.g. mouse #4, Fig. 4B). After nine weeks, four out of the five mice showed statistically significant differences in these bioluminescence signals between control sides and miR-199b-5p counter sides (Fig. 4C). Taken together, these data show that miR-199b-5p can impair tumor formation *in vivo* in athymic nude/nude mice. The xenograft tumors from mouse #5 and mouse #4 were explanted and analyzed for miR-199b-5p and HES1 expression and evaluated histopathologically (Text S1, Fig. S4E–H).

**Figure 5:**
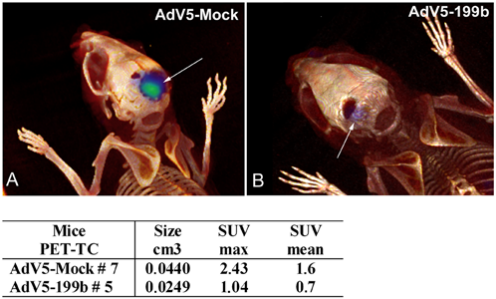
High-resolution molecular imaging of xenografted MB cells injected into the cerebellum. PET-CT fusion images of AdV5-Mock#7 (A) and AdV5-199b#5 (B) mice at 12 weeks from surgery and injection, with 3D volume rendering and simultaneous display of areas of FLT uptake (blue–green, white arrows). A wider bone defect in the occipito-parietal portion of the skull is apparent in the AdV5-Mock#7 mouse. Below: table of measurements (cm^3^) of tumor mass undertaken with PET-CT acquisition (as described in Text S1).

To further investigate the ability of miR-199b-5p to regulate MB growth, we injected the 199bSC1 stable clone orthotopically into the fourth ventricle of nude mice of 5 weeks of age (Fig. 4D, E). After four weeks of *in vivo* non-invasive monitoring tumor growth by BLI, in mice injected with the 199bSC1 clone the tumor growth was considerably lower than that observed in the control (CTL)-cells-injected side. As further confirmation of these effects, we also injected the CTL cells infected with an adenovirus coding for miR-199b-5p: in agreement with the previous findings, these mice also showed reduced BLI after 4 weeks (Fig. 4F). Further BLI acquisitions after 8 weeks from implantation of the cells are shown in Figure 4G. At this time, two mice were sacrificed and further analysed for histopathology. Hematoxylin-eosine staining of frozen tissues showed tumor mass in the cerebellum of AdV5-Mock#2 and AdV5-199b#3 injected animals. Serial parallel frozen histological sections were examined by fluorescence microscopy for endogenous green fluorescence protein (GFP) expressed by adenovirus-infected cells. Then, we evaluated HES1 protein expression by immunohistochemistry staining of other paraffin-embedded tissues, using an anti-HES1 antibody. Overall, we assessed the levels of persistence of adenovirus expression in infected cells, as the down-regulation of HES1 expression due to miR199b carrying the adenovirus expression, thus following tumor growth over time by BLI see Figure 4G–H, and antibodies staining Figure S4L–M (Hes1, Ki67, Gabra6, Nestin, Math-3, see details in Text S1 information). Then, two additional nude mice (AdV5-Mock#7; AdV5-199b#5) underwent PET-CT studies at 12 weeks post-injection, to assess tumor proliferative activity (Fig. 5A, B). Additional BLI data together with two movies (Movie S1 and Movie S2) showing the 3D reconstruction of the orthotopic engraftment are described in the Text S1 information and showed in Figure S6. These analyses showed significant reduction of tumor mass in the AdV5-199b#5 animal, as compared to the AdV5-Mock#7 control mice, with PET-CT analyses also providing tumor volumes (0.024 cm^3^ versus 0.044 cm^3^, respectively), as described in Text S1. Overall, these data indicate a beneficial effect of over-expression of miR199b-5p, as a negative regulator of tumor growth of MB cells in this orthotopic xenograft nude-mouse model.

### Expression of miR-199b-5p in human medulloblastoma tumors

**Figure 6:**
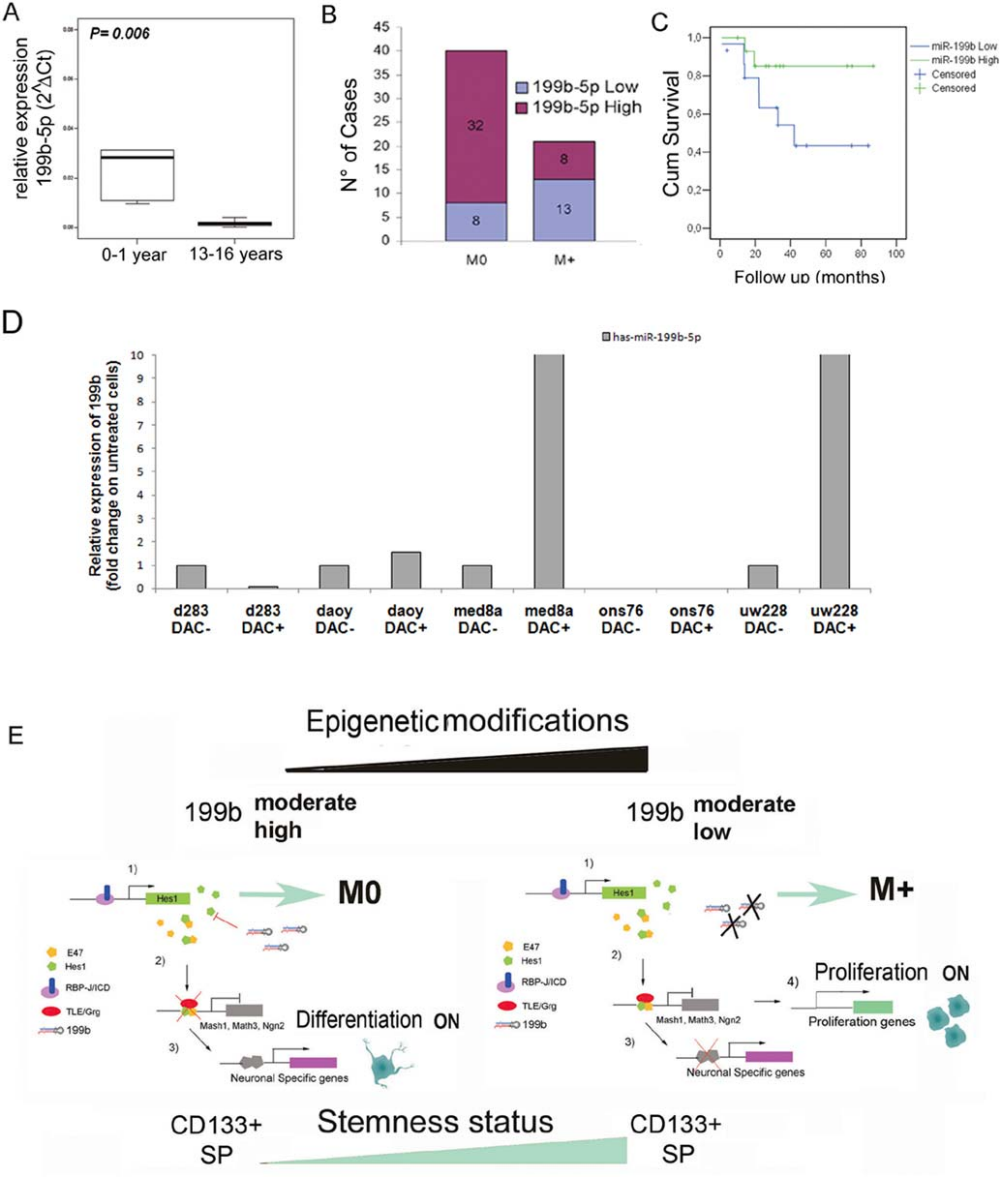
Expression levels of miR199b in human cerebellum and in tumors, and the correlation with prognosis. A) Box-plot of expression levels of miR-199b-5p in healthy human cerebella in the two age ranges indicated. MiR-199b-5p showed higher expression in explants from younger controls (Mann-Whitney test, *P* = 0.006). B) MiR-199b-5p highly expressing cases are mostly M0 *P* = 0.001 (Pearson Chi-Square test). C) Kaplan-Meier survival estimates comparing patients with low *versus* high (relative to median) levels of miR-199b-5p expression (45 patients with available follow-up data). D) MiR-199b-5p expression by real-time PCR in a panel of five MB cell lines untreated or treated with 5-Aza-C (DAC−/+); the de-methylation induced miR-199b-5p transcription in two cell lines: Med8a and UW228. E) The interplay between miR-199b-5p and the Notch pathway. M0 and M+ patients show different expression levels of miR-199b-5p, which could be driven by an epigenetic mechanism. Left side: Under conditions of induced miR-199b-5p expression (1), the levels of HES1 decrease, relieving the inhibition on the neurogenic bHLHs (2). This in turn promotes a neurogenic programme and a decrease in proliferation, SP and CD133+ cells (3). Right side: Under conditions of low miR-199b-5p expression, the levels of HES1 are higher (1), repressing the pro-neural bHLH genes, with a consequent increase in proliferation, SP and an enlargement of the CD133+ compartment (4).

To determine whether miR-199b-5p is effectively expressed in healthy human pediatric cerebella, we used 13 control samples obtained from the NICHD Brain and Tissue Bank for Developmental Disorders, at the University of Maryland, USA. We measured miR-199b-5p expression, comparing five cerebellum samples obtained from 0–1-year-old children with six from 13–16-year-old children (Fig. 6A). MiR-199b-5p showed greater expression in the explants from the younger healthy controls (Mann-Whitney test, *P* = 0.006).

To determine whether miR-199b-5p expression has a role in human MB, samples from a cohort of 61 MB patients were analysed (see Experimental Procedures). Indeed, it has already been shown that HES1 protein levels correlate with negative outcome in MB patients [24]. The whole patient population (n = 61) was then divided into two groups, as low *versus* high miR-199b-5p expression, based on the overall median. The distribution of miR-199b-5p expression between non-metastatic (M0) and metastatic (M1, M2 and M3) cases showed that miR-199b-5p expression in the non-metastatic cases was significantly higher than in the metastatic cases (*P* = 0.001, Pearson Chi-Square test; Fig. 6B). In the subset of patients where follow-up information was available (n = 45), the survival curve for the patients who expressed miR-199b at high levels showed a positive trend, with better overall survival than the low-expressing patients. However, the log-rank test of the Kaplan-Meier curves did not show a significant difference (*P* = 0.182; Fig. 6C), probably due to the limited number of patients with long-term follow-up.

These data showing down-regulation of miR-199b-5p in metastatic MBs indicates a mechanism of silencing through epigenetic or genetic alterations. We thus tested expression of miR-199b-5p by real-time PCR in a panel of MB cell lines following induction of de-methylation with 5-aza-deoxycytidine (Fig. 6D). Indeed, two cell lines (Med8a and UW228) showed significant up-regulation of miR-199b-5p, thus supporting the hypothesis of epigenetic control of miR-199b-5p expression. Further studies need to be performed to identify this tuned regulation mechanism of action through epigenetic inactivation of miR199b-5p expression during tumor development.

## Discussion

The interconnections between the signaling pathways altered in MB remain largely unknown, and it is still difficult to define specific patient stratification according to risk classes that would lower therapy-linked mortality and morbidity. In the present study, we have identified a mechanism of HES1 gene regulation via the miRNA miR-199b-5p. HES1 is a key regulator of cerebellum development, as indicated by recent findings that treatment of cerebellar granule cells with Shh increases the levels of HES1 [20]. This suggests that HES1 is a transcriptional target of both the Shh and Notch2 signaling cascades.

We report here a novel level of regulation of HES1 expression that is driven by miR-199b-5p, which binds to the HES1 3′UTR and leads to unproductive translation. Of note, we have also investigated the actions of miR-199b-5p on a reporter construct fused with the 3′UTR of NHLH2, cyclin L1 and Nanog (Text S1, Fig. S2D). Among the predicted targets for miR-199b-5p, which were obtained using two different algorithms (Text S1, Table S1), these genes were possible candidates related to the phenotype obtained after miR-199b over-expression. Nonetheless, we did not obtain down-regulation of reporter activity when tested under the same conditions where we obtained effects on HES1 3′UTR. Furthermore, the stable 199bSC1 clone has a GSK3-β protein level comparable to the control. Of note, when analyzed according to the Pita algorithm, the value of ΔΔG for the binding of miR-199b-5p to the 3′UTR of GSK3-β is very high, indicating poor accessibility to the 3′UTR [41] (see Text S1, Table S1). Recently it has also been shown that miR-199a*, which has the same sequence as miR-199b-5p, is involved in caspase activation by potentially acting through Met proto-oncogene down-regulation [42]. It will be of interest for future studies to also determine the down-regulation of this target by miR-199b, even if it appears unlikely because of the lack of induction of apoptosis after miR-199b over-expression in our cellular systems.

The analysis of the over-expressing miR-199b-5p clones suggested that miR-199b-5p can impair the proliferation and engrafting potential of MB cells. Indeed loss of Cyclin D1 in over-expressing miR-199b-5p clones are a reminder of the similar effects seen in *Ccnd1*–/– mice, which have decreased early GNP proliferation and early ataxia as a consequence of a delay in acquiring normal cerebellar function, thus affecting progression of the pre-neoplastic lesions to MBs [43].

Our data are also in agreement with the *in-vivo* data reported from murine cells genetically knocked down for *Hes1*. While the effects of miR-199b-5p could be explained by triggering of apoptosis, our cytofluorimetric assays suggested that impairment of MB cell proliferation is not linked to extensive apoptosis of cells over-expressing miR-199b-5p (see cell cycle assay on Fig. 1A). This is in agreement with reported data for neural progenitors obtained from *Hes1*−/− mice, where apoptotic indications were only seen in restricted cell types [22].

The correlation that we show between miR-199b-5p and tumor M stage indicates that miR-199b-5p expression levels can be investigated concomitant with tumor resection. This will allow identification of a subset of patients who should develop an aggressive tumor and have future metastasis formation, noting that disseminated disease is the most powerful independent factor associated with poor survival. Despite the correlation with M status, the over-expression of miR-199b-5p did not lead to a decrease in cell motility of Daoy cells (Text S1, Fig. S2E). This may well be due to the extremely high motility of Daoy cells, indicating that to overcome this phenotype, a further step beyond regulation of the Notch pathway needs to take place. However, of note, the expression of PDGFR-B and SPARC, two genes recently correlated with metastatic MB [44], [45], is reduced in the presence of over-expression of miR-199b-5p, as we show in Text S1, Fig. S5D and describe in Text S1.

In the light of our data on the regulation of the Notch pathway via miR-199b-5p modulation, it will be of interest to focus future studies on the role of Notch-regulated genes in metastatic MB patients. Additionally, developing mouse models that better reproduce the human disease is an important task. Within this scenario, the findings that the homozygous Smo/Smo mouse model has leptomeningeal spread confirms involvement of the Notch pathway in regulation of MB growth [25], [46]. As shown in Fig. 6 and Text S1, Table S2, we show great variability in the miR-199b-5p expression levels, which might be due to genetic chromosomal alterations or epigenetic silencing in a subset of patients with worse outcome. Our data on a panel of MB cell lines (see Fig. 6D) support this last hypothesis. The reason why some of the MB cell lines do not respond to the treatment with increasing miR-199b could be due to genetic loss of the gene itself, or to a strict cell-type regulation of miR-199b expression, reflecting the different origins of the cell lines.

At present, there is growing interest in the elucidation of the mechanisms that confer unique properties to the ‘cancer stem cells’ [47]. Recently, it was shown that glioblastoma CD133+ cells have a better chance of survival after ionizing radiation, through the induction of the repair of damaged DNA [48]. Indeed, Daoy cells expressing the CD133 antigen are radio-resistant, thus supporting the hypothesis that Daoy cells represent a model for the study of a tumor stem-cell compartment [49]. Here miR-199b-5p can influence this side population and the CD133+ population of Daoy cells, namely the cancer stem cells.

Together with CD133, we also evaluated the expression levels of several cancer stem-cell genes that have recently been shown to be correlated with the aggressiveness of solid tumors; as shown in Text S1, Figure S5 (A–D) and table S3, the expression of these genes is in agreement with an overall reduction in the ‘stemness’ phenotype of Daoy cells treated with *N*-[*N*-(3,5-difluorophenacetyl)-l-alanyl]-*S*-phenylglycine *t*-butylester (DAPT). This prevents activation of the Notch response (see Text S1, Materials), thus blocking the presenilin-secretase complex and enhancing miR199b-5p expression [50], [51]. To date, this is the first report that expression of an miRNA can deplete this tumor cell compartment, indicating an interesting therapeutic approach for the targeting of these cells in brain tumors.

There is a strong indication from published series that children with medulloblastoma presenting at over 3 years of age benefit greatly from radiotherapy, but the treatment of younger children remains a challenge. The absence of significant differences in survival rates between patients with total or subtotal excision of MB supports the view that the total excision of MB can be avoided when the risk for potential neurological deficits is high. Recent trials have demonstrated a beneficial effect of chemotherapy, although this was not universally seen [52], [53]. Here, we envisage the use and delivery of miR199b-5p *in situ* into the cerebellum of MB-affected children under 3 years of age (and positive to HES1), to thus impair the maintenance of the tumor-initiating CD133+ cancer cells. Combined with chemotherapy, and with the potential to abrogate radiotherapy and avoid brain damage due to total tumor excision, this should provide an overall improvement in the present treatments for MB. Thus we foresee the possibility of treating MBs with the use of encapsulated stable nucleic acid lipid particles (SNALP). These have been demonstrated to be effective in non-human primates for systemic delivery, and would by-passing the blood-brain barrier using encapsulated nano-particles containing agomir 199b-5p molecules [54]. How these gene-therapy approaches will progress further will be an issue for future pre-clinical animal studies.

As illustrated in our model (Fig. 6E), we picture two different expression levels of miR-199b-5p in M0 and M+ patients, which might be due to epigenetic regulation during carcinogenesis. In our ‘moderate high’ model, an increase in miR-199b-5p expression represses HES1, which then leads to an increase in pro-neural bHLHs gene expression, driving the cell towards differentiation processes. In the ‘moderate low’ model, miR-199b-5p expression is lowered due to epigenetic control mechanisms, and then HES1 is over-expressed, leading to cell proliferation and induction of the SP and hence an increase in CD133+ cells. As for many other transcription factors, HES1 is a point of integration between and among different signal transduction pathways, and its balance of expression determines fundamental cell decisions, such as whether or not to start a differentiation program. With this scenario, miR-199b-5p can be seen as part of the complex Notch signal-transduction pathway, as a fine tuner of the levels of expression of the HES1 bHLH transcription factor. These phenomena could be considered to occur in a variety of tissues and cancers where an activated Notch pathway is involved.

## Materials and Methods

### Ethics Statement

All animal work have been conducted according to relevant national and international guidelines. Approval was obtained by Institutional Animal Care and Ethical Committee at CEINGE - University of Naples Federico II, Protocol #13 - 08/01/2007, and the Italian Ministry of Health; Dipartimento Sanità Pubblica Veterinaria D.L. 116/92, confirming that all experiments conform to the relevant regulatory standards.

### Flow cytometry analysis

The flow cytometry analysis of S-phase fractions and cell-cycle kinetics were carried out using a FACSCalibur (Becton Dickinson, San Jose, CA) using CELL Quest version 3.3 software. The CD133 studies were carried out using the same instrument, with antibodies from Miltenyi Biotec (Auburn, CA), according to the manufacturer instructions. In brief, the cells were blocked in Fc receptor blocking reagent and incubated with an anti-CD133/1 (AC133)-phycoerythrin antibody (Miltenyi Biotec), for 10 min in the dark at 4°C. The cells were then washed and resuspended in PBS. Cells expressing higher levels of CD133 than the immunoglobulin G (IgG) controls were considered positive. For analyses of the side populations, cells (10^6^/mL) were incubated with Hoechst 33342 (5 µmol/L; Sigma) for 90 min at 37°C in EMEM containing 2% FBS. To ensure the correct identification of the side population cells, the cells were incubated as above with the addition of 50 µM verapamil (Sigma).

### In-vivo imaging via stable Daoy luciferase clones

Stable Daoy cell clones were generated by transfecting a luciferase-expressing vector (the firefly luciferase gene cloned in the plentiV5 vector; Invitrogen) into the stable 199bSC1 clone or into the control empty-vector clone. Regarding xenograft subcutaneous implantations, 100,000 viable 199b-Luc1 and Ctl-Luc-4 cells were mixed with a 1∶1 PBS∶matrigel solution (BD Biosciences, San Jose, CA). Six-week-old female athymic mice were previously anesthetised using avertin (Sigma) as a 3% solution in tert-amyl alcohol (Fisher), at a dose of 3 mg per 10 g body weight. then injected s.c. with a total volume of 0.1 mL of matrigel PBS cell suspension, into each flank. Regarding xenograft orthotopic delivery into cerebellum a protocol described since by Schabet M. et al., [55], was used to deliver MB cells after microsurgical exposure of the atlanto-occipital membrane, 50 µl of cerebrospinal fluid was aspirated through a 27 G needle and replaced by 50 µl of DMEM containing 100,000 DAOY medulloblastoma cells. The wound was sutured with 3-0 polyglactin 910, and 4-0 silk. The mice were imaged after the implantation of the cells, with tumor growth monitored by weekly bioluminescence acquisitions (BLI) using an IVIS 3D Illumina Imaging System (Xenogen Corp. Alameda, CA). For the acquisitions, the mice were isofluorane-anaesthetised i.p., injected with 100 µL D-luciferin (15 mg/mL stock) per 10 g body weight, and imaged for 30 s, 10 min after luciferin injection; four acquisitions per mouse were made (ventral, dorsal and each flank). To quantify the bioluminescence, the integrated fluxes of photons (photons per s) within each area of interest were determined using the Living Images Software Package 3.0 (Xenogen-Caliper). The emission data from the start of tumor growth were collected for at least 6 weeks, and then were normalized to the bioluminescence on the injection day. Calliper measurements of tumor sizes were made weekly, along the long and short axes, and estimations of their volumes were made using the formula: width^2^×length×0.52.

Animals were monitored daily by weights and neurological examinations to determine their good standard status of health. Those animals showing sign of health sufferance were promptly sacrificed. On day 63, after cell inoculation, all animals were sacrificed with an overdose of ketanest and xylazin.

### Animal preparation and PET/CT imaging

The mice were kept in a ventilated cage (26°C) for 1 h prior to imaging studies. Anesthesia was performed with intraperitoneal administration of a mixture of ketamine (100 mg/kg) and xylazine (10 mg/kg) (injection volume, 100 µl/10 g). PET was performed 1 h after administration of 3′-deoxy-3′-[^18^F]fluorothymidine ([^18^F]FLT), a marker of tumor proliferation (50 µL; 7.4 MBq; scan time, 18 min), in a lateral caudal vein, using an animal PET scanner (GE Healthcare eXplore Vista, FWHM 1.6 mm). High resolution CT studies (GE Healtcare eXplore Locus; spatial resolution, 45 µm) were performed within 24 h from the PET.

### Data Analysis

Maximum (SUVmax) and mean (SUVmean) standardized uptake values (SUVs) were calculated from the PET studies (SUV = tissue activity (MBq/cc)/[injected dose (MBq)/body weight (g)]). The PET/CT images were post-processed to obtain multiplanar reconstructions (MPRs), maximum intensity projections (MIPs), 3D volume rendering, and fusion images, using Osirix 3.3 (MAC OS 10.5 operating system). Additional 3D reconstructions were obtained using MicroView (GE eXplore Locus).

Lesion volumes were calculated from PET data using in-house-developed software (based on IDL, ITT Vis Inc), by summarizing all spatially connected voxels with SUV >50% SUVmax. Lesion profiles defined with these procedures were used for ROI-based comparison between AdV5-Mock and AdV5-199b.

## Supporting Information

Text S1 Supporting Information
(0.12 MB DOC)

Figure S1 A) Representative Western blots from transient transfection of HEK293 cells with expression constructs for miR-199b-5p and miR-199a. MiR-199b-5p over-expression lowered the levels of the endogenous HES1 protein; in contrast, miR-199a induced an increase in HES1 levels 48 h after transfection. Right: Quantification through densitometric analyses. B) Representative Western blot from transient transfection of D283 cells with the expression construct for miR-199b-5p and using an empty vector. Again, miR-199b-5p over-expression lowered the levels of the endogenous HES1 protein. Below a quantification through densitometric analyses. C) D283 and SH-SY5Y cells express similar levels of miR-199b-5p and miR-124a, with the latter known to be preferentially expressed in the central nervous system. In contrast, the D283Med cells showed considerably lower levels of miR-199b-5p. The data shown are means±SD from two independent experiments, each carried out in triplicate. D) Representative MiR-199b-5p expression profiles across a panel of human tissues (Ambion); miR-199b-5p was expressed to different degrees, with relatively high expression in the duodenum, lymph nodes, lung, skeletal muscle, right ventricle (highest), kidney, total heart and thyroid. MiR-199b-5p effects on the 3′UTR of its putative target gene, HES1. E–F) Luciferase activity from a reporter vector containing wild-type HES1 3′UTR and HES1 3′UTR mutated in the miR-199b binding site, co-transfected or not with an expression vector for miR-199b-5p. The luciferase from the wild-type 3′UTR activity was reduced by 50% with miR-199b-5p expression, while 2′-O-methyl-oligoribonucleotide (2-OM; 400 nM) blocks this effect. There is no effect with the miR-199b-5p together with the HES1 3′UTR mutated in the binding site. A representative experiment is shown where the data are means±SD from three replicates in HEK293 and Daoy cell lines respectively. G–I) Pre-miR-199 was cloned and transfected into Daoy cells, and three stable clones were evaluated for HES1 expression by qRT PCR and Western blotting. There was a significant decrease in HES1 protein levels, as revealed using an anti-HES1 polyclonal antibody; the decrease was also revealed by densitometry analysis (G; right panel) H) A 2′-O-methyl-oligoribonucleotide (2-OM) directed against miR-199b-5p was transfected into the stable 199bSC1 clone, with a representative Western blot and the quantification by densitometric analysis showing restored HES1 expression. The quantification data shown are means±SD from two independent experiments, each carried out in triplicate.
(7.18 MB TIF)

Figure S2 Mmu-miR-199b expression in mouse embryonic cerebellum and regulation of other potential targets by human miR-199b-5p. A) Mmu-miR-199b in situ mRNA expression is detectable at E14.5 and in newborn mouse (p0) cerebellum. The staining was diffuse, and in all areas of the cerebellum; expression decreased from E14.5 to p0. Mmu-miR-124a (a brain specific miRNAs) was used as control. Left: magnification, 50×; Right: magnification, 200×. B) Quantification of decrease in mmu-miR-199b expression during mouse development and differentiation of the cerebellum. The levels of expression of mature miR-199b-5p are given relative to let-7A, with the data shown as means±SD from two independent experiments, each carried out in triplicates C) Representative Western blot showing the protein levels of GSK3-Beta which is predicted to be a target, in the higher expressing miR-199b-5p stable clone. GSK3-Beta levels were not down-regulated, and were instead slightly increased, as is clear from the quantification by densitometric analysis shown in the right panel, where the data shown are means±SD from two independent experiments, each carried out in triplicate D) MiR-199b-5p is predicted to bind other UTRs (see Supporting Table S1). When miR-199b-5p over-expression in Daoy cells was examined for down-regulation of productive translation from a reporter gene carrying the full-length 3′UTRs indicated, none of them were seen to be affected. The data shown are means±SD from two independent experiments, each carried out in triplicate. E) Cell motility assays comparing the 199bSC1 cell line with the Daoy empty vector CTR (control) line using a Boyden chamber system (0.5% FBS as chemoattractant). The higher motility cells were fixed and hematoxylin stained and counted under the microscope. The data shown are means±SD from two independent experiments, each carried out in triplicate, and they indicate no differences between control (CTR) and the 199bSC1 cell line. F) Proliferation assay (MTS) of D283MED and ONS76 cells over-expressing miR-199b-5p after transient transfections, as indicated. The D283MED cells transfected with 199b-5p (blue diamonds) show appreciable reduction in cell proliferation, as compared to the control, empty vector, transfection (green triangles). ONS76 cells show a higher proliferation rate that is nonetheless affected by the 199b-5p transfectant (compare orange crosses of empty vector transfection to purple squares). The data shown are means±SD from two independent experiments, each carried out in triplicate G) Silencing of endogenous expression of miR-199b-5p via transfection of a 2-O-methyl oligoribonucleotide antisense (2-OM-a) leads to an increase in Daoy cells proliferation, probably relieving the control of endogenous miR-199b-5p on HES1 3′UTR. The data shown are means±SD from two independent experiments, each carried out in triplicate.
(8.54 MB TIF)

Figure S3 FACS analysis for the role of miR-199b-5p on the Daoy cell side population. A, C, E) Decrease in the SP cells of the Daoy 199bSC1 stable clone, as determined by Hoechst 33342 staining. B, D, F) Addition of verapamil to force dye incorporation also in the SP cells. The Daoy cells showed a 5.2% fraction of SP cells, while the stable 199bSC1 and 199bMC1 clones over-expressing miR-199b-5p do not show significant levels of SP cells (0.2%, 0.4%, respectively)
(8.54 MB TIF)

Figure S4 Creation of bioluminescent Daoy cells over-expressing miR-199b-5p for in-vivo studies. The Daoy stable clone with the empty vector (Ctl) and the Daoy stable clone over-expressing miR-199b-5p (199b) were transfected with a luciferase expression vector (Luc), generating, respectively, the Ctl-Luc#4 clone, and the199bLuc-1 and 199b-Luc-3 clones. These were all evaluated for their expression of bioluminescence when incubated with the enzyme substrate, luciferin. A) Light emission of serial dilutions of the cells indicated in a microplate. The bioluminescence signal was acquired by an IVIS 200 Imaging System (Xenogen Corp. Alameda, CA). B) Correlation between cell number and photon emission. The 199b-Luc1 and Ctl-Luc#4 clones where used for the in-vivo studies, with the data shown as means±SD from two independent experiments C) Representative data of the expression of miR-199b-5p in the luciferase clones, relative to the luciferase clone carrying the empty vector. D) Representative immunoblot analysis showing lowered expression of HES1 in the 199b-Luc clones, compared to the empty-vector clone. E, F) Hematoxylin and eosin staining of xenografts derived from mouse #4: the control-injected side shows the xenograft infiltrating the muscle tissue, while the 199b-injected side does not. G, H) Nestin is a marker of neuroblasts, and its expression correlates with a lesser differentiated state: the xenograft from the mouse #4 199b-injected side shows a decrease in Nestin positivity. Nestin staining was perfomed by immunohistochemistry with a polyclonal antibody (Abcam). Magnification, 100×. I) Representative gene expression levels of the transgene miR-199b-5p in the tumor explant from mouse #5. The over-expression of miR-199b-5p was lost. L, M, N) Immunohistochemistry with anti-HES1, anti-KI67, anti-Gabra6, anti-Nestin, anti-Math3 antibodies and hematoxylin staining of the xenografts from the cerebellum of AdV5-Mock and AdV5-199b mice. Significant expression of Hes1, Ki67, Nestin and Math3 proteins is seen in the AdV5-Mock cerebellum tumoral tissue, while very low Hes1, Ki67 Nestin and Math3 expression is seen in the AdV5-199b tumor cells in the cerebellum. Differences of expression of Gabra6 are barely observed between in the AdV5-Mock cerebellum tumoral tissue and AdV5-199b mice. Left: magnification, 25×; Right: magnification, 100×.
(9.53 MB TIF)

Figure S5 Over-expression of endogenous miR199b upon DAPT treatment induces down-regulation of genes involved to embryonic stem cells and cancer stem cells in the Daoy cell line. A) Representative data of a time course experiment of DAPT treatment of Daoy cell lines (6 h, 12 h, 24 h) with media supplemented and replaced every 4 h, and fold of expression of miR199b determined using quantitive real time detection, relative to time 0. B) Representative Western blot showing HES1 down-regulation after 12 h of induction of DAPT in the Daoy cell line. C) Relative gene expression levels of CD133, c-Myc Oct4, KFL5, Nanog, TCF7L1, HMGA1, HMGB3, ZIC1, MYBL2, TEAD4, ILF3 in the Daoy cell line with and without DAPT treatment for 12 h. D) Relative gene expression of PDGFR-A, PDGFR-B and SPARC in the Daoy cell line with and without DAPT treatment for 12 h The primers used and their DDct values of relative expression using real-time cDNA quantitative detection, are listed in Supporting Table S3.
(0.47 MB PDF)

Figure S6 Mir-199-b-5p interferes with the engraftment potential of Daoy cells injected into mouse cerebellum. A) In-vivo bioluminescence analyses using IVIS 3D of 199b-Luc1 mouse xenografted with Daoy Luc1 cell line overexpressing miR-199b by stable clone analyses. (see mouse #3; see additional data in Fig. 4F, main text). The mouse was scanned once a week for a total of 8 weeks. B) BLI measurements (photon/sec/cm-2) from mice carrying cells infected with ADV5-199b show reductions to week 8. C) Images taken by IVIS Spectrum at 560 nm and 660 nm to generate the topography of the subject. Clt-Luc-4 AdV5-Mock #5 xenografted mice shown on a 3D axis (x,y,z), with the extension of the tumor burden shown in comparison with a digital mouse atlas that enabled the display of the 3D skeleton and organs on the 3D reconstruction, using Living Images software. (See also Movie S1 and Figure 4 panel F for BLI fold changes values) D) As described above, analyses are taken on Ctl-luc AdV5 199b #5 xenografted mice (See also Movie S2 and Figure 4 panel F for BLI fold changes values).
(8.30 MB TIF)

Table S1 Cancer related targets of miR-199b-5p. MiRanda and Pita algorithms were applied to the selected “cancer-related” miR-199b-5p targets. The gene targets predicted by both of these algorithms are listed, including: MIRANDA score, P values, Pita analyses with sequence matches and Delta-Delta G values.
(0.04 MB DOC)

Table S2 Patients characteristics recruited for the study. A) In columns Age at diagnosis in month, Follow-up time in months, State at last news, Mestatasis (M) stage, Histology, Relative expression of miR-199b-5p compared to the level of (U6) and 2^−Delta^ Ct values. B) Normal healthy cerebellum mRNA from the Brain and tissue Bank, University of Maryland, Baltimore (USA) were used for this study. In columns, ID of material, age in year and relative expression of miR-199b-5p compared to the level of (U6) and 2^−Delta^ Ct values.
(0.15 MB DOC)

Table S3 Stem and cancer stem genes, and genes associated with MB tumor development in Daoy cell lines over-expressing endogenous miR-199b-5p under treatment with DAPT. The genes involved in stem cell biology selected for the study of their expression after over-expression of miR-199b-5p. The Unigene ID and sequence of primers used for real-time PCR are shown, the 2^−Delta^ Ct values are obtained from the analysis of Daoy cells treated with DAPT for 12 h and analysed with the 7700 Real Time TaqMan Applied Biosystem.
(0.05 MB DOC)

Movie S1 (26.01 MB MOV)

Movie S2 (21.61 MB MOV)
