# Supplementary material for: MicroRNA-199b-5p Impairs Cancer Stem Cells through Negative Regulation of HES1 in Medulloblastoma
Source: PLoS One. 2009 Mar 24;4(3):e4998. doi: 10.1371/journal.pone.0004998 (PMC2656623; doi:10.1371/journal.pone.0004998)
Supplement: Text S1 — Supporting Information (0.12 MB DOC) [file pone.0004998.s001.doc]

**Supporting Information**

Through target prediction analyses, miR-199a-5p and miR-199b-5p were seen to potentially target HES1, with high score values. To determine whether this potential binding translated into efficient HES1 regulation in living cells, we transfected both miRNAs in the HEK-293 cell line (Supporting Fig. S1 A, B). We cloned the pre-miRNAs into two constructs where expression was driven by the CMV promoter. After 48 h of transient transfection, miR-199b-5p did indeed down-regulate HES1 expression. At this stage, we could exclude effects of miR-199a-5p on HES1 3’UTR in different cell types too.

We then evaluated the expression levels of mature miR-199b-5p using a real-time approach (see Experimental Procedures) in Daoy and D283MED cells (Supporting Fig. S1C). MiR-199b-5p showed low expression levels in SH-SY5Y cells, a neuroblastoma cell line, while the Daoy cells expressed more miR-199b-5p. There were also differences in the expression levels of the two miRNAs in the D283MED MB cell line, which was derived from the ascites fluid of a metastatic MB patient, unlike the Daoy cell line [1]. Here, the expression of miR-199b-5p was 10^4^-fold lower when compared to the Daoy cells, potentially reflecting the different tumor cell type origins [2]. The expression of miR-199a* was also evaluated (data not shown), which is also known as miR-199a-3p, and which has recently been linked to the MET proto-oncogene [3]. After over-expression of pre-miR-199a, we did not see over-expression of miR-199a* in Daoy cells. This is probably due to a specific processing of pre-miR-199b-5p towards the production of miR-199a (data not shown). MiR-199b-5p expression was also evaluated in normal human tissues (Supporting Fig. S1D): its expression was high in the duodenum and lymph nodes, with lower expression seen in adult (whole) brain and cortex.

To confirm the effects of miR-199b-5p on different MB cell lines, we transiently transfected D283MED cells and evaluated the down-regulation of HES1 by Western blotting. As shown in Supporting Figure S1 G, the over-expression of miR-199b-5p led to a decrease in the HES1 protein levels. This effect on HES1 protein translates into a reduction in cell proliferation, as evaluated by the MTS assay (see Supporting Fig. S2 F). In this case, we compared D283MED and ONS76 cell lines transiently transfected with miR-199b-5p; in both cases we saw decreased proliferation, as compared to mock-transfected cells.

So, we asked if miR-199b-5p is involved in the control of cell proliferation at endogenous levels. To answer this, we used a 2-O-methyl-oligoribonucleotide directed against the mature miR-199b-5p sequence (199b-OM), to decrease its endogenous levels. As Daoy cells showed the highest amounts of miR-199b-5p among the MB cell lines tested (data not shown), they were transfected with 199b-OM (Supporting Fig. S2 G). The cells showed a higher proliferation rate *in vitro*, compared to the proliferation rate of the Daoy cells transfected with a control OM (a scrambled 2-O-methyl oligoribonucleotide).

**Mmu-miR-199b-5p expression in developing cerebellum**

To address the question as to how mir199 is expressed during cerebellum development, we made use of the mmu-miR-199b-5p murine homolog and assayed for its expression in the developing mouse cerebellum. We applied the methodology developed for the use of locked nucleic acid probes labeled with digoxygenin (DIG). Embryos at E14.5 and newborn mouse brains at P0 were used in *in-situ* hybridization experiments, as shown in Supporting Figure S2A. The expression of mmu-miR-199b-5p was compared to that of miR-124a as the control, which is known to be an abundant miRNAs in the central nervous system. The expression of mmu-miR-199b-5p was more diffuse, as compared to miR-124a. Also, mmu-miR-199b-5p expression decreased with development of the cerebellum, while miR-124a retained its expression in the internal granule cell layer (IGL) and in Purkinje neurons. Then, the expression of mmu-miR-199b-5p was evaluated in the mouse cerebellum at different developmental stages, by measuring the levels of mature miRNA and through its expression analyses using real-time PCR. The data showed that even if mmu-miR-199b-5p was expressed at lower levels compared to miR-124a, mmu-miR-199b-5p expression is regulated in development, decreasing from embryonic days 16.5 to the adult developed mouse cerebellum (Supporting Fig. S2 B).

**MiR-199b-5p regulation of other potential targets.**

Among the predicted targets of miR-199b-5p, there is the kinase GSK3-β, a protein involved in signal transduction from the Shh and Wnt pathways, and thus a crucial kinase in the pathways regulating cerebellar cell homeostasis. For this reason, we evaluated the ability of miR-199b-5p to down-regulate GSK3-β using the already obtained stable 199bSC1clone. This clone did not show any significant reductions in the levels of the GSK3-β protein (Supporting Fig. S2 C). This effect can be explained according to the 3’UTR of GSK3-β not being accessible for binding with miR-199b-5p, as is indeed suggested by the high ΔΔG evaluated by the Pita algorithm (see Supporting Table S1; [4]). We also selected three other genes to be tested for their potential regulation by miR-199b-5p: Nanog, NHLH2 and cyclin L1. When down-regulated, these genes could lead to a phenotype similar to that seen in the stable miR-199b-5p clones. We did not detect any down-regulatory effects of miR-199b-5p on these selected gene 3’UTRs (see Supporting Figure S2 D). More recently, it has been shown that miR-199a* regulates the MET proto-oncogene [3]. Since it shares the same sequence as miR-199b-5p, it will be of interest to check the levels of this proto-oncogene in our analyses, which will be an issue of future studies.

**Creation of bioluminescent Daoy cells over-expressing miR-199b-5p for *in-vivo* studies.**

Luciferase-based, *in-vivo* imaging has emerged recently as a powerful tool to investigate the engrafting potential of tumor cells in orthotopic animal models [5]. To determine the role of miR-199b-5p in an *in-vivo* tumor model, we stabilized the 199bSC1 and control Daoy clones with an expression vector carrying luciferase cDNA. The clones were tested to assess their luciferase expression levels (see Supporting Fig. S4 A, B): these *in-vitro* experiments showed high correlations between cell numbers and bioluminescent measurements. Thus, this luciferase expression allowed the non-invasive imaging of tumor-associated bioluminescence and quantification of tumor growth. The stable clones obtained were validated for their retention of the parental phenotype, in terms of both miR-199b-5p and HES1 expression, as illustrated in Supporting Figure S4 C, D. The xenograft from mouse #4 was explanted and processed to determine its morphology, using haematoxylin and eosin staining. The tumors formed on the control side were more invasive and aggressive than those formed on the miR-199b-5p-injected side, as can be seen by the invasion of the former into the muscle tissue (Supporting Figure S4 E; black arrows). The staining with an antibody directed against Nestin revealed that the tumor formed on the miR-199b-5p side showed less cells with the characteristic of neural progenitors, in agreement with Notch pathway down-regulation (Supporting Figure S4 G, H) [6]. The explants from mouse #5, which formed tumors on both the 199b- and control-injected sides, showed a loss of expression of the transgene (miR-199b-5p), which could be responsible for the recovery of tumor potential (see Supporting Figure S4 I).

To further characterize the ability of miR-199b-5p to counteract tumor growth *in vivo*, we established orthotopic implants of Ctl-Luc1 and 199b-Luc4 cells. In addition, we also injected wild-type Daoy cells that were previously infected with a recombinant adenovirus expressing miR-199b-5p (AdV5-199b), followed by *in-vivo* imaging of bioluminescence (BLI). We used both 199b-Luc1 as controls, and the Ctl-Luc4 clone infected with an AdV5-mock virus. The results 4 weeks from injection are shown in Figure 4F, with more BLI analyses of the mice shown at 4 and 8 weeks (Figure 4G and Figure S6).

**The Daoy cell line treated with DAPT shows increased endogenous expression of miR-199b and an embryonic stem-cell-like gene expression signature.**

To determine whether we could increase endogenous levels of miR-199b-5p, we treated Daoy cell lines with *N*-[*N*-(3,5-difluorophenacetyl)-l-alanyl]-*S*-phenylglycine *t*-butylester (DAPT, Sigma-Aldrich), which efficiently blocks the presenilin-secretase complex, and as a consequence, efficiently prevents activation of the Notch response [7,8]. As shown in Supporting Figure S5 A, we found that upon treatment with DAPT, the level of miR-199b-5p increased by 9-fold at 12 h. This activation was then followed by rapid inhibition of its expression, within the 24 h of the treatment. These data were then supported by verifying the levels of HES1 using Western blot analyses (Supporting Figure S5 B), thus indicating that following miR-199b-5p over-expression, inhibition of the HES1 protein levels was seen. These data prompted us to determine to what extent HES1 loss and miR199 over-expression can influence the gene expression signature of genes involved in embryonic stem (ES) and cancer stem cells upon DAPT treatment.

Following the results presented in one recent study [9], we applied real-time quantitative detection for the expression profiles of the genes (Top-8 list) that were enriched and associated with embryonic stem (ES) cell identity, and the ‘stemness’ properties of several solid tumors, including breast, bladder, and glioma. Here, we selected markers for stem-cell genes (MB and glioblastoma), such as CD133 and c-Myc, and then genes that are key regulators of ES cell identity: Oct4, KFL5 and Nanog. We also looked at genes associated with adult stem cell/ progenitor function, ES and tumor cell proliferation and/or cancer progression. According to these criteria, the gene list comprised: CD133, c-Myc, Oct4, KFL5, Nanog, TCF7L1, HMGA1, HMGB3, ZIC1, MYBL2, TEAD4 and ILF3. As shown in Supporting Figure S5 C, the expression of CD133 and c-Myc genes was down-regulated in the Daoy cell line induced with DAPT. Then the expression levels of the Oct4, Nanog, KFL5, TCF7L1, HMGA1, HMGB3, ZIC1, MYBL2 and TEAD4 genes were also seen to be down-regulated in Daoy DAPT-induced over-expression of miR-199b-5p, primers and raw 2^DCt values are listed in Tab S3. This thus confirmed that miR-199b-5p up-regulation is concurrent with inhibition of the Notch pathway, which regulates stem-cell populations, as seen by those selected markers that are already known to be involved ‘directly’ or ‘indirectly’ in this phenomena. At present, it remains to be determined to what extent the over-expression of miR-199b-5p can be modulated by inhibition of the Notch pathway. These questions will be an issue for future studies.

Additional studies have demonstrated over-expression of PDGFR-A, PDGFR-B and SPARC genes in metastatic MB patients and in MB cell lines [10,11]. We thus also analysed the expression of PDGFR-A, PDGFR-B and SPARC genes in the Daoy cell line model as presented above, with its up-regulation of endogenous miR199b upon treatment with DAPT. As shown in Supporting Figure S5D, the expression of PDGFR-B and SPARC were down-regulated in the Daoy cell line treated with DAPT, as compared with vehicle only treatment, while low up-regulation of PDGFR-A also seen. At this time, it remains to be determined if this low increase in the expression of PDGFR-A through the RAS/MAPK pathway will be sufficient to drive the metastatic potential of MB cells overexpressing miR-199b-5p; this is an opposite effect to that we obtained by verifying expression of miR199b-5p in tumors. These results are also somehow contradictory with the data in the literature, although in framework of an erratum [11], the over-expression of PDGFR-A found in MB metastatic tumors was miss-judged due to an incorrect sequence and probe annotation by GenBank and Affymetrix: although GenBank sequence J03278 includes the complete coding region of PDGFR-B, the locus was given as ‘PDGFRA’, and Affymetrix probe 1771 was annotated as ‘J03278 HUMPDGFRA Human platelet-derived growth factor (PDGF) receptor mRNA’. Within these new reported data, PDGFR-B was seen to be up-regulated in metastatic MB tumors. These latest results are in line with what we have seen in our Daoy cells lines, where over-expression of miR-199b-5p upon DAPT treatment induced down-regulation of expression of PDGFR-B and SPARC. This in turn correlates with the loss of expression of miR199b-5p in metastatic patients, and definitively strengthens the importance of miR-199b-5p expression and its involvement in MB cancer development, impairing their metastatic potential. Finally, the data here presented confirmed the role of miR-199b-5p over-expression by linking its main function to inhibiting ES and cancer stem cell maintenance operated by genes already involved in these mechanisms of action.

Very recently, a specific expression pattern from childhood MB patients showed nine miRNAs, which included miR-199b-5p, also clustered with ErbB2- and/or c-Myc-over-expressing tumors [12]. In particular, they showed higher expression of miR-135a and b, miR-10b, miR-125b, miR-153, and mir199b-5p in ErB2-positive patients. Overall, those results from the literature strengthen further the importance of mir199b-5p, the expression of which was found up-regulated in MB tumors [12]. Of note Gilbertson et al. [13] have show that ErbB2-HER4-positive tumors behave as independent prognostic markers in childhood MB, and in particular Erb-2 is a worse prognostic marker given its negative survival effect in MB. Ferretti et al. [12] shows that overexpression of mir199b-5p was occurring in 12 cases out of 33 MB tumors analysed, while in our report analyses we found that lower expression mir199b-5p is correlated to tumor analysed mainly M+ patients (see Fig.6 B). Ferretti et al. associated then mir199b-5p overexpression to aggressiveness of Erb-2-positive cohorts using clustering analyses. We think these reported data are different from our results because of the different tumor source and the different statistical correlations studies applied (Hierarchical clustering analyses versus Pearson Chi-Square test). We think our analyses are more accurate because of the number of samples used and the use of three independent tumors samples sources, thus improving the power of our statistical association analyses. In summary, we used for the evaluation of the expression of mir199b-5p an Italian, a French and a Canadian tumor bank, totaling 45 samples analyzed with the entire follow-up information here presented (see Table 2 Supplemental).

**High-resolution molecular imaging by small-animal PET and SPECT-CT analyses.**

***Animal preparation and PET/CT imaging***

The mice were kept in a ventilated cage (26 °C) for 1 h prior to imaging studies. Anesthesia was performed with intraperitoneal administration of a mixture of ketamine (100 mg/kg) and xylazine (10 mg/kg) (injection volume, 100 μl/ 10 g). PET was performed 1 h after administration of 3'-deoxy-3'-[^18^F]fluorothymidine ([^18^F]FLT), a marker of tumor proliferation (50 μL; 7.4 MBq; scan time, 18 min), in a lateral caudal vein, using an animal PET scanner (GE Healthcare eXplore Vista, FWHM 1.6mm). High resolution CT studies (GE Healtcare eXplore Locus; spatial resolution, 45μm) were performed within 24 h from the PET.

***Data Analysis***

Maximum (SUVmax) and mean (SUVmean) standardized uptake values (SUVs) were calculated from the PET studies (SUV = tissue activity (MBq/cc) / [injected dose (MBq)/ body weight (g)]). The PET/CT images were post-processed to obtain multiplanar reconstructions (MPRs), maximum intensity projections (MIPs), 3D volume rendering, and fusion images, using Osirix 3.3 (MAC OS 10.5 operating system). Additional 3D reconstructions were obtained using MicroView (GE eXplore Locus).

Lesion volumes were calculated from PET data using in-house-developed software (based on IDL, ITT Vis Inc), by summarizing all spatially connected voxels with SUV >50% SUVmax. Lesion profiles defined with these procedures were used for ROI-based comparison between AdV5-Mock and AdV5-199b.

The results presented in Figure 5 (main text) showed a tumor mass at the level of a wide skull defect at implant site potentially due to a high level of tumorigenesis occurring within the skull and high level of cartilaginous-bone degeneration with corresponding FLT uptake at PET. In this PET-CT analyses, we confirmed the presence of the development an enlarged tumor in the cerebellum of the control mice (AdV5-Mock#7), compared to the mice xenografted with over-expressing cells miR-199b-5p (AdV5-199p#5).

**Supporting Experimental Procedures**

***Standard cell culture.***

The human Daoy and D283-MED MB cell lines were obtained from American Type Culture Collection (ATCC, Manassas, VA) and were maintained in Eagle’s minimum essential medium (EMEM; Sigma) supplemented with 10% fetal bovine serum (FBS), 10 U/ml penicillin and 0.1 mg/ml streptomycin (Celbio Pero, Milan, Italy). HEK-293 and SH-5YSY cells were maintained in Dulbecco’s modified Eagle’s medium (DMEM; Sigma) supplemented with 10% FBS, 10 U/ml penicillin and 0.1 mg/ml streptomycin.

***Transfection of Daoy cells and luciferase assays***

To determine the activity of miR-199b-5p on repression of the luciferase-3’UTR fused constructs, the pREYZO-miR-199b-5p construct and the pRL-CMV-3’UTR target construct were co-transfected into HEK293 cells at a ratio of 10:1, using the Polyfect reagent (Qiagen) together with the pGL3 basic vector, in the absence and presence of 2-OM (400 nM). Luciferase activities were analysed using a Dual Luciferase Reporter Assay system (Promega , Madison, WI).

***AZA treatment***

Five MB cell lines (d283, Daoy, med8a, ons76, uw228) were grown in DMEM supplemented with 10% FBS and antibiotics. Upon reaching 85% confluence, the media was spiked with 5-aza-deoxycytidine (AZA) (Sigma) to a final concentration of 5 μM. Over 72 h the media was replaced daily with fresh AZA-enhanced medium. RNA was extracted using a standard phenol (TRIzol)-chloroform protocol.

***Vector cloning***

Pre-miR-199b was cloned in a pcDNA3 modified vector (pREYZO) (D’angelo et al., 2004) using *EcoRI–XhoI* restriction sites. HES1 and GSK-3β target-binding sites (3’UTRs) were cloned in tandem in the 3’UTR of the pRL-cytomegalovirus (CMV) vector (Promega, Madison, WI) downstream of the coding region of *Renilla luciferase* (RL) in the XbaI site. The HES1 3’UTR (including the 199b binding site) was amplified from genomic DNA with the following primers: HES1_3'UTR as aaaatctagacagttcgaagacataaaagcc and HES1_3'UTR as aaaatctagaaacgcagtgtcaccttcc, and was cloned in the TK vector (Promega, Madison, WI) upstream of the firefly luciferase gene, in the *XbaI* sites. HES1 full-length cDNA was obtained with a standard RT-PCR approach from a Daoy cDNA.

***Site-directed mutagenesis***

The site-directed mutagenesis of the miR-199b-5p binding site in HES1 3’UTR was generated according to the manufacturer protocol of the QuikChange site-directed mutagenesis kit (Stratagene, La Jolla, CA). The following primer was used for mutagenesis (only sense sequence is given, and the mutated nucleotides within the consensus sequence are underlined): HES1 3’UTR sense AACAGGAACTTGAATATTTGTAGAGAAGAGGACTTT.

***TaqMan miRNAs assay***

*Reverse transcriptase reactions*

The reverse transcriptase (RT) reactions contained 40 ng RNA sample, 50 nM stem–loop RT primer, 1x RT buffer (P/N:4319981, Applied Biosystems), 0.25 mM each of dNTPs, 3.33 U/ml MultiScribe RT (P/N: 4319983, Applied Biosystems) and 0.25 U/ml RNase inhibitor (P/N: N8080119; Applied Biosystems). The 15 μl reactions were incubated in an Applied Biosystems 9700 Thermocycler for 30 min at 16 °C, 30 min at 42 °C, 5 min at 85 °C, and then held at 4 °C.

# Real-time PCR

Real-time PCR was performed using a standard TaqMan PCR kit protocol on an Applied Biosystems 7900HT Sequence Detection System. The 20 μl PCR mix included 2 µl RT product, 10 µl TaqMan Universal PCR Master Mix (Applied Biosystems), 0.2 mM TaqMan probe. The reactions were incubated in a 96-well plate at 95 °C for 10 min, followed by 60 cycles of 95 °C for 15 s and 60 °C for 1 min.

***RNA isolation, cDNA preparation and real-time quantitative PCR***

Total RNA was extracted from the cell lines using the Trizol reagent (Invitrogen). Synthesis of cDNA from total RNA (4 µg) was performed using a Super Script II First Strand Kit (Invitrogen). Real-time quantitative PCR was performed using standard protocols with an Applied Biosystems ABI PRISM 7900HT Sequence Detection system. To calculate the relative gene expression of the miR-199b-5p precursor, the relative amounts of each pre-miRNA were normalized to U6 miRNA; the other genes were normalized using the β-actin gene. Real-time PCR primers for each gene were designed using Primer Express software version 2.0 (Applied Biosystems). The primer sequences are available upon request.

***Western blotting***

Thirty µg cytosolic or nuclear lysates were loaded onto 12% polyacrilamide gels, and blotted onto polyvinylidene difluoride membranes (PVDF; BioRad, Milan, Italy). The membranes were then incubated with *anti-HES1* (kind gift of Dr.Tetsuo Sudo TORAY Industries, Tebiro Kamakura, Japan), a polyclonal rabbit antibody (1:500), an anti-GSK3-β (B&D Bioscience) monoclonal antibody (1:2500), an anti-c-Myc polyclonal rabbit antibody (1:200) (Santa Cruz Biotechnology, Santa Cruz, CA, USA), an anti-GABRA6 antibody (Santa Cruz Biotechnology, Santa Cruz, USA) (1:200), an anti-MATH3 antibody (Santa Cruz Biotechnology, Santa Cruz, USA) (1:200) and an anti-cyclin D1 (1:200) polyclonal rabbit antibody (Cell Signaling). An anti-β-actin antibody (1:1.000) (Sigma) was used as the control for equal loading of cytosolic lysates and a polyclonal anti-β-laminin antibody (1:50) (Santa Cruz Biotechnology, Santa Cruz, CA, USA ) for equal loading of nuclear lysates.

***Soft-agar assays***

Daoy cells and the stable 199b clone were seeded in 1 ml EMEM supplemented with 2% FBS, containing 0.3% agarose. Cultures were maintained for two weeks and refreshed with EMEM supplemented with 2% FBS twice per week. Colonies of more than 100 μm in diameter were counted under a Leica (Nussloch, Germany) DC500 compound microscope.

***Proliferation assay MTS***

Cell proliferation was determined using the CellTiter96 AQueous Non-Radioactive Cell Proliferation Assay Kit (Promega Madison, WI). The cells were seeded into 96-well plates at a density of 1x 10^3^ cells/well in EMEM without or with FBS. After 0, 24, 48 and 72 h, MTS ([3-(4,5-dimethylthiazol-2-yl)-5-(3-carboxymethoxyphenyl)-2-(4-sulfophenyl)-2H-tetrazolium]) and PMS (phenazine methosulfate) were added to each well and after 4 h the absorbance of each well was measured at 490 nm (OD_490_) using Multilabel counter Victor^3^ (Perkin Elmer). Each condition was performed with five wells and each experiment was repeated twice.

***Immunohistochemistry***

Unmasking was performed in 10 mM citrate buffer, pH 6, at 97 °C for 45 min. Blocking was performed with Antibody Diluent with Background Reducing Components (Dako Cytomation) for 30 min at room temperature; the polyclonal anti-HES1(1:1000), anti-Nestin (1:1000), anti-GABRA6 (1:100), anti-MATH3 (1:100) antibodies were used at overnight at 4 °C. The signals were revealed using Kit LSAB DAKO, for 15 min (Biotin), and Kit LSAB DAKO, for 15 min (Streptavidina), at room temperature.

DAB was from DakoCytomation, and the slides were mounted and examined under a Leica (Nussloch, Germany) DC500 compound microscope.

***Locked nucleic acid in-situ hybridization***

Dried slides from murine embryonal brain or human multiple tissue arrays were fixed in 4% paraformaldehyde for 10 min at room temperature, and washed twice for 3 min in 1x PBS at room temperature. During the washing steps, the acetylation solution was freshly prepared, containing: 0.01% triethanolamine and 0.0025 mM acetic anhydride in diethylenepyrocarbonate (DEPC ) water. The slides were then placed in a beaker of acetylation solution and stirred gently for 10 min. The slides were then subjected to proteinase K treatment at 5 μg/m1 in DEPC-treated water, followed by three washes for 3 min in 1x PBS at room temperature, and pre-hybridized for 6 h at room temperature with hybridization solution. Hybridization was with 150 μl denaturizing hybridization, with 1 pM of the locked nucleic acid DIG-labelled probe (Exiqon, miRCURY). Hybridization was performed at 60 °C overnight. Slides were soaked in pre-warmed 60 °C 5x SSC and the coverslips were carefully removed; the slides were then incubated in 0.2x SSC at 60 °C for 1 h, then incubated in a 0.1 M Tris, pH 7.5, 0.15 M NaCl at room temperature for 10 min. The surplus solution was removed and the slides placed in a humidified chamber; 500 μl of blocking solution (1% FCS in 0.1 M Tris, pH 7.5, 0.15 M NaCl) was placed on the slides for an incubation of 1 h at room temperature. The anti-DIG-alkaline phosphatase antibody was diluted 1:2,000 in blocking solution and incubated at 4 °C overnight. The slides were washed three times in 0.1 M Tris, pH 7.5, 0.15 M NaCl, and then equilibrated in 0.1 M Tris, pH 9.5, 0.1 M NaCl, 50 mM MgCl. The slides were stained with BCIP/NTB (Sigma), dehydrated, and mounted; images are acquired under a Leica (Nussloch, Germany) DC500 compound microscope.

***DAPT treatment of Daoy cell line***

The human Daoy cell line was obtained from American Type Culture Collection (ATCC, Manassas, VA) and maintained in EMEM (Sigma Aldrich, Milan, Italy) supplemented with 10% fetal bovine serum (FBS) (Celbio Pero, Milan, Italy), 10 U/ml penicillin and 0.1 mg/ml streptomycin (Celbio Pero, Milan, Italy). HEK-293 and SH-5YSY cell were maintained in DMEM (Sigma) supplemented with 10% FBS (Celbio), 10 U/ml penicillin and 0.1 mg/ml streptomycin (Celbio).

*N*-[*N*-(3,5-difluorophenacetyl)-l-alanyl]-*S*-phenylglycine *t*-butylester (DAPT (Sigma-Aldrich) treatment of the Daoy cells was performed as follows: the cells were plated and allowed to grow overnight in medium containing 10% FBS. The next morning, the medium was replaced with medium containing 10 μM DAPT and replaced each 4 h with this medium. The expression assays was performed after 0, 6, 12, 24 h of treatment of miR199b, using control experiments including the corresponding volume of DMSO as vehicle.

***In-vitro cell-motility assay***

The control DAOY cell line and the stable DAOY-199bSC1 clone were used in the ‘cell-motility’ assays. Cell migration assays were performed with 8-µm pore size Transwells (Costar). The cells were suspended by trypsinization, washed, and resuspended in 100 µl serum-free DMEM containing 0.1% BSA (5x 10^4^ cells) and placed in the upper chamber of the Transwells. The lower chamber was filled with 600 µl serum-free DMEM supplemented with 0.5% FBS, as the chemo-attractor. The cells were allowed to migrate for 1 h at 37 °C. Cells migrating to the lower side of the polycarbonate membrane were fixed with 2.5% glutaraldehyde and stained with Hematoxylin solution (Sigma). The cells were counted, and the analysis performed on three Transwells for each condition, and each experiment was repeated twice.

***Patient samples***

A total of 61 cases of MB were collected from three different centres: 12 surgical MB specimens were from the Department of Neurosurgery, Santobono Hospital, Naples, Italy; 18 were from the Institute Curie, Paris, France; and 31 were from The Hospital for Sick Children, Toronto, Canada. The characteristics of the patients are given in Supporting Table S2. Informed consent was obtained before the analysis of the tumor samples. All of the specimens were obtained at the time of diagnosis, prior to radiation or chemotherapy, and 29 were subjected to histopathological review according to the WHO criteria [14], the M status information was available for all of the patients. Control human tissues were obtained from the NICHD Brain and Tissue Bank for Developmental Disorders at the University of Maryland, Baltimore, Maryland. Total RNA was extracted using Trizol reagent (Invitrogen), as described above for cell lines, and analyzed for miR-199b-5p expression levels by the TaqMan MicroRNAs Assay. The miR-199b-5p expression levels were divided into two groups (low and high), according to the median, and were compared between metastatic and non-metastatic groups using the Pearson Chi-Square test. Survival analyses were obtained through SPSS software, using a Log-rank test to ascertain significance. Statistical significance was established at a *P* value of 0.05.

***Adenovirus construction***

The miR199b sequence was 5’-XhoI, 3’-HindI III directed cloned into the VQ Ad5CMV K-NpA shuttle vector, supplied from ViraQuest Inc., Innovative Adenovirus Technologies and Reagents, which provided its recombination and 199b adenovirus construction. They also supplied the control backbone E3 Luciferase virus, generated from a VQ Ad5CMVeGFP plasmid.

***Adenoviral infection***

Infection with recombinant viruses was accomplished by exposing cells to adenovirus in 500 μl complete cell culture medium for 1 h, followed by addition of other medium. We used GFP-expressing by the adenovirus (AdV MOCK and AdV 199b) as a control when determining transfection efficiency.

***Heterotransplantion into right ventricle of cerebellum of SCID mice***

To establish intracerebellar xenograft models, 6-to-8-week-old SCID mice were anesthetized with Tribromoethanol (Avertin®) (50 mg/kg); after this, a small skin incision (1 mm) was made and a burr hole (0.7 mm in diameter) created with a microsurgical drill (Fine Science Tools, Foster City, CA). Daoy Luc cells infected with adenovirus mock, adenovirus 199b and 199b LUC1 stable clones (10^5^) were suspended in 5 μL PBS and injected slowly through the burr hole into the right cerebellar hemisphere (stereotactic coordinates from bregma anteroposterior 5.5 mm; right lateral 2.1 mm; dorsoventral 5.0 mm) using a 10-AL, 26-gauge Hamilton Gastight 1701 syringe needle that was inserted perpendicular to the cranial surface. The animals were monitored weekly by bioluminescence to evaluate tumor growth for 8 weeks.

***3D acquisition***

IVIS Spectrum: this system acquires a photographic and a structured light image, and two or more bioluminescent images at different wavelengths (560 to 660 nm), and generates the surface topography (mesh) of the subject. Modifying specific user-modifiable DLIT algorithm parameters (for example, analysis wavelengths, source spectrum, and tissue properties), we can reconstruct the position, geometry and strength of the luminescent sources in the subject. The Living Image® software provides digital mouse atlases that allow the display of a 3D skeleton and organs on the 3D reconstruction.

***Animal PET and SPECT-CT***

The mice were then anesthetized with 2.5% isofluorane and kept under 1% isofluorane anesthesia throughout all of the imaging procedures. The [^18^F]FDG and [^18^F]Fluoride PET imaging was used to examine tumor-cell-induced metabolic and skeletal changes, respectively. The images were acquired with three bed positions (10 min per bed position, for a total of 30 min) and an axial resolution of 1.2 mm, using an Explorer-Vista small animal PET-CT system (GE Healthcare).

**Supporting Information References**

1. Friedman HS, Burger PC, Bigner SH, Trojanowski JQ, Wikstrand CJ, et al. (1985) Establishment and characterization of the human medulloblastoma cell line and transplantable xenograft D283 Med. J Neuropathol Exp Neurol 44: 592-605.

2. Jacobsen PF, Jenkyn DJ, Papadimitriou JM (1985) Establishment of a human medulloblastoma cell line and its heterotransplantation into nude mice. J Neuropathol Exp Neurol 44: 472-485.

3. Kim S, Lee UJ, Kim MN, Lee EJ, Kim JY, et al. (2008) MicroRNA miR-199a* regulates the MET proto-oncogene and the downstream extracellular signal-regulated kinase 2 (ERK2). J Biol Chem 283: 18158-18166.

4. Kertesz M, Iovino N, Unnerstall U, Gaul U, Segal E (2007) The role of site accessibility in microRNA target recognition. Nat Genet 39: 1278-1284.

5. Rubin JB, Kung AL, Klein RS, Chan JA, Sun Y, et al. (2003) A small-molecule antagonist of CXCR4 inhibits intracranial growth of primary brain tumors. Proc Natl Acad Sci U S A 100: 13513-13518.

6. Fan X, Matsui W, Khaki L, Stearns D, Chun J, et al. (2006) Notch pathway inhibition depletes stem-like cells and blocks engraftment in embryonal brain tumors. Cancer Res 66: 7445-7452.

7. Dovey HF, John V, Anderson JP, Chen LZ, de Saint Andrieu P, et al. (2001) Functional gamma-secretase inhibitors reduce beta-amyloid peptide levels in brain. J Neurochem 76: 173-181.

8. Geling A, Steiner H, Willem M, Bally-Cuif L, Haass C (2002) A gamma-secretase inhibitor blocks Notch signaling in vivo and causes a severe neurogenic phenotype in zebrafish. EMBO Rep 3: 688-694.

9. Ben-Porath I, Thomson MW, Carey VJ, Ge R, Bell GW, et al. (2008) An embryonic stem cell-like gene expression signature in poorly differentiated aggressive human tumors. Nat Genet 40: 499-507.

10. MacDonald TJ, Brown KM, LaFleur B, Peterson K, Lawlor C, et al. (2001) Expression profiling of medulloblastoma: PDGFRA and the RAS/MAPK pathway as therapeutic targets for metastatic disease. Nat Genet 29: 143-152.

11. Gilbertson RJ, Clifford SC (2003) PDGFRB is overexpressed in metastatic medulloblastoma. Nat Genet 35: 197-198.

12. Ferretti E, De Smaele E, Po A, Di Marcotullio L, Tosi E, et al. (2008) MicroRNA profiling in human medulloblastoma. Int J Cancer 124: 568-577.

13. Gilbertson RJ, Pearson AD, Perry RH, Jaros E, Kelly PJ (1995) Prognostic significance of the c-erbB-2 oncogene product in childhood medulloblastoma. Br J Cancer 71: 473-477.

14. Giangaspero F, Wellek S, Masuoka J, Gessi M, Kleihues P, et al. (2006) Stratification of medulloblastoma on the basis of histopathological grading. Acta Neuropathol 112: 5-12.
