# Supplementary figures and images for: MicroRNA-199b-5p Impairs Cancer Stem Cells through Negative Regulation of HES1 in Medulloblastoma
Source: PLoS One. 2009 Mar 24;4(3):e4998. doi: 10.1371/journal.pone.0004998 (PMC2656623; doi:10.1371/journal.pone.0004998)

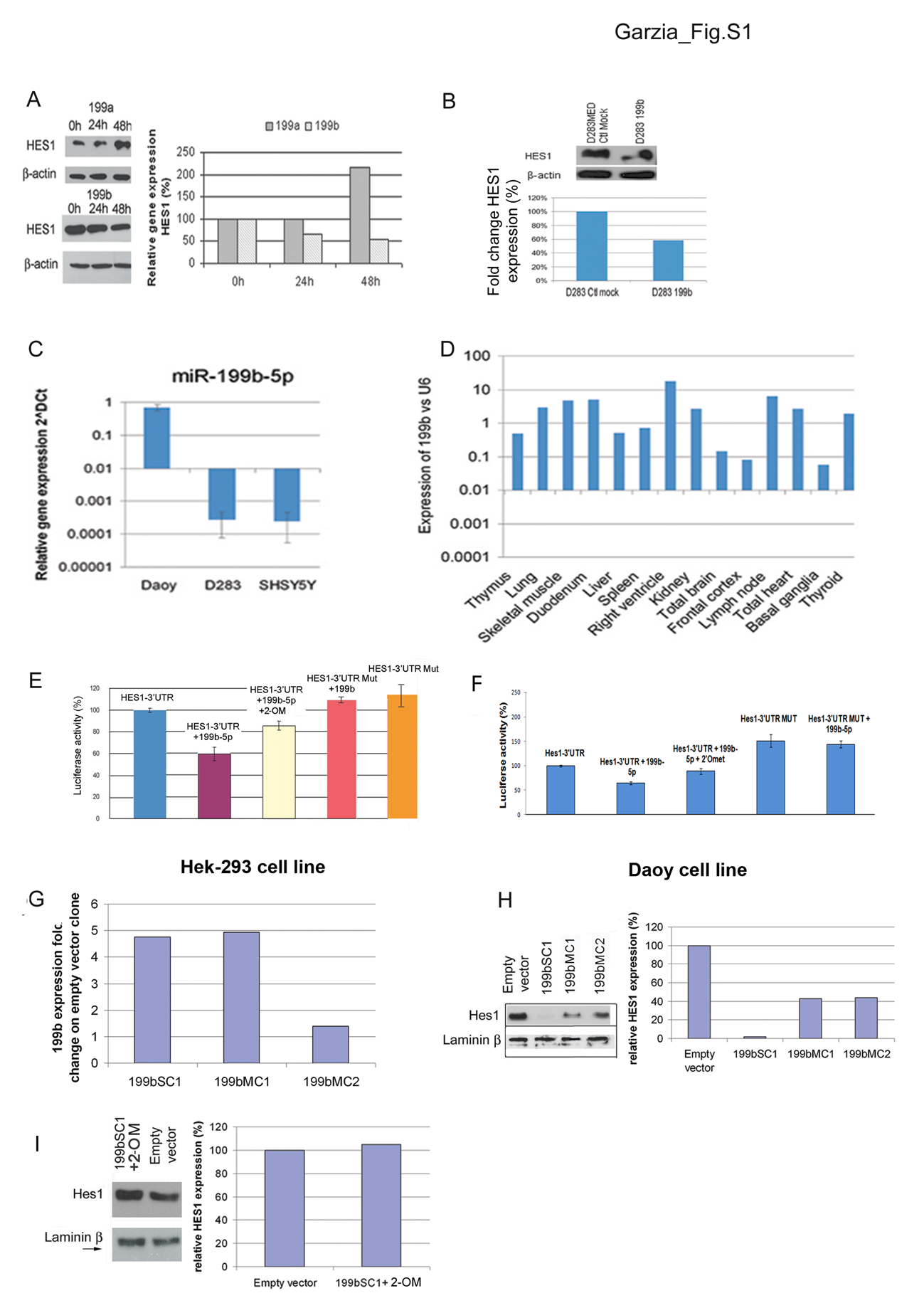

Supplement: Figure S1 — A) Representative Western blots from transient transfection of HEK293 cells with expression constructs for miR-199b-5p and miR-199a. MiR-199b-5p over-expression lowered the levels of the endogenous HES1 protein; in contrast, miR-199a induced an increase in HES1 levels 48 h after transfection. Right: Quantification through densitometric analyses. B) Representative Western blot from transient transfection of D283 cells with the expression construct for miR-199b-5p and using an empty vector. Again, miR-199b-5p over-expression lowered the levels of the endogenous HES1 protein. Below a quantification through densitometric analyses. C) D283 and SH-SY5Y cells express similar levels of miR-199b-5p and miR-124a, with the latter known to be preferentially expressed in the central nervous system. In contrast, the D283Med cells showed considerably lower levels of miR-199b-5p. The data shown are means±SD from two independent experiments, each carried out in triplicate. D) Representative MiR-199b-5p expression profiles across a panel of human tissues (Ambion); miR-199b-5p was expressed to different degrees, with relatively high expression in the duodenum, lymph nodes, lung, skeletal muscle, right ventricle (highest), kidney, total heart and thyroid. MiR-199b-5p effects on the 3′UTR of its putative target gene, HES1. E–F) Luciferase activity from a reporter vector containing wild-type HES1 3′UTR and HES1 3′UTR mutated in the miR-199b binding site, co-transfected or not with an expression vector for miR-199b-5p. The luciferase from the wild-type 3′UTR activity was reduced by 50% with miR-199b-5p expression, while 2′-O-methyl-oligoribonucleotide (2-OM; 400 nM) blocks this effect. There is no effect with the miR-199b-5p together with the HES1 3′UTR mutated in the binding site. A representative experiment is shown where the data are means±SD from three replicates in HEK293 and Daoy cell lines respectively. G–I) Pre-miR-199 was cloned and transfected into Daoy cells, and three stable c [file pone.0004998.s002.tif]

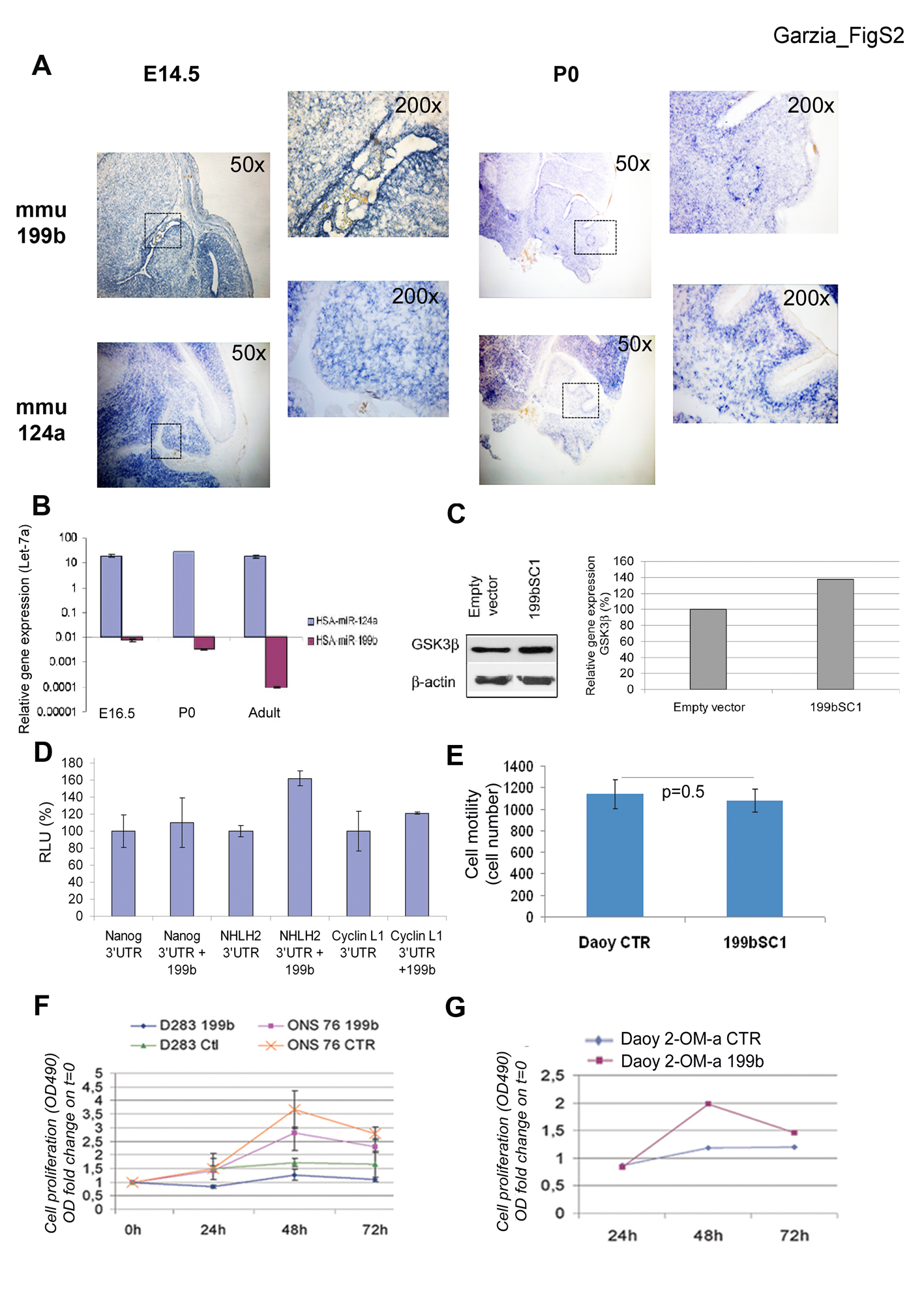

Supplement: Figure S2 — Mmu-miR-199b expression in mouse embryonic cerebellum and regulation of other potential targets by human miR-199b-5p. A) Mmu-miR-199b in situ mRNA expression is detectable at E14.5 and in newborn mouse (p0) cerebellum. The staining was diffuse, and in all areas of the cerebellum; expression decreased from E14.5 to p0. Mmu-miR-124a (a brain specific miRNAs) was used as control. Left: magnification, 50×; Right: magnification, 200×. B) Quantification of decrease in mmu-miR-199b expression during mouse development and differentiation of the cerebellum. The levels of expression of mature miR-199b-5p are given relative to let-7A, with the data shown as means±SD from two independent experiments, each carried out in triplicates C) Representative Western blot showing the protein levels of GSK3-Beta which is predicted to be a target, in the higher expressing miR-199b-5p stable clone. GSK3-Beta levels were not down-regulated, and were instead slightly increased, as is clear from the quantification by densitometric analysis shown in the right panel, where the data shown are means±SD from two independent experiments, each carried out in triplicate D) MiR-199b-5p is predicted to bind other UTRs (see Supporting Table S1). When miR-199b-5p over-expression in Daoy cells was examined for down-regulation of productive translation from a reporter gene carrying the full-length 3′UTRs indicated, none of them were seen to be affected. The data shown are means±SD from two independent experiments, each carried out in triplicate. E) Cell motility assays comparing the 199bSC1 cell line with the Daoy empty vector CTR (control) line using a Boyden chamber system (0.5% FBS as chemoattractant). The higher motility cells were fixed and hematoxylin stained and counted under the microscope. The data shown are means±SD from two independent experiments, each carried out in triplicate, and they indicate no differences between control (CTR) and the 199bSC1 cell line. F) Proliferation assay (MTS) of D283 [file pone.0004998.s003.tif]

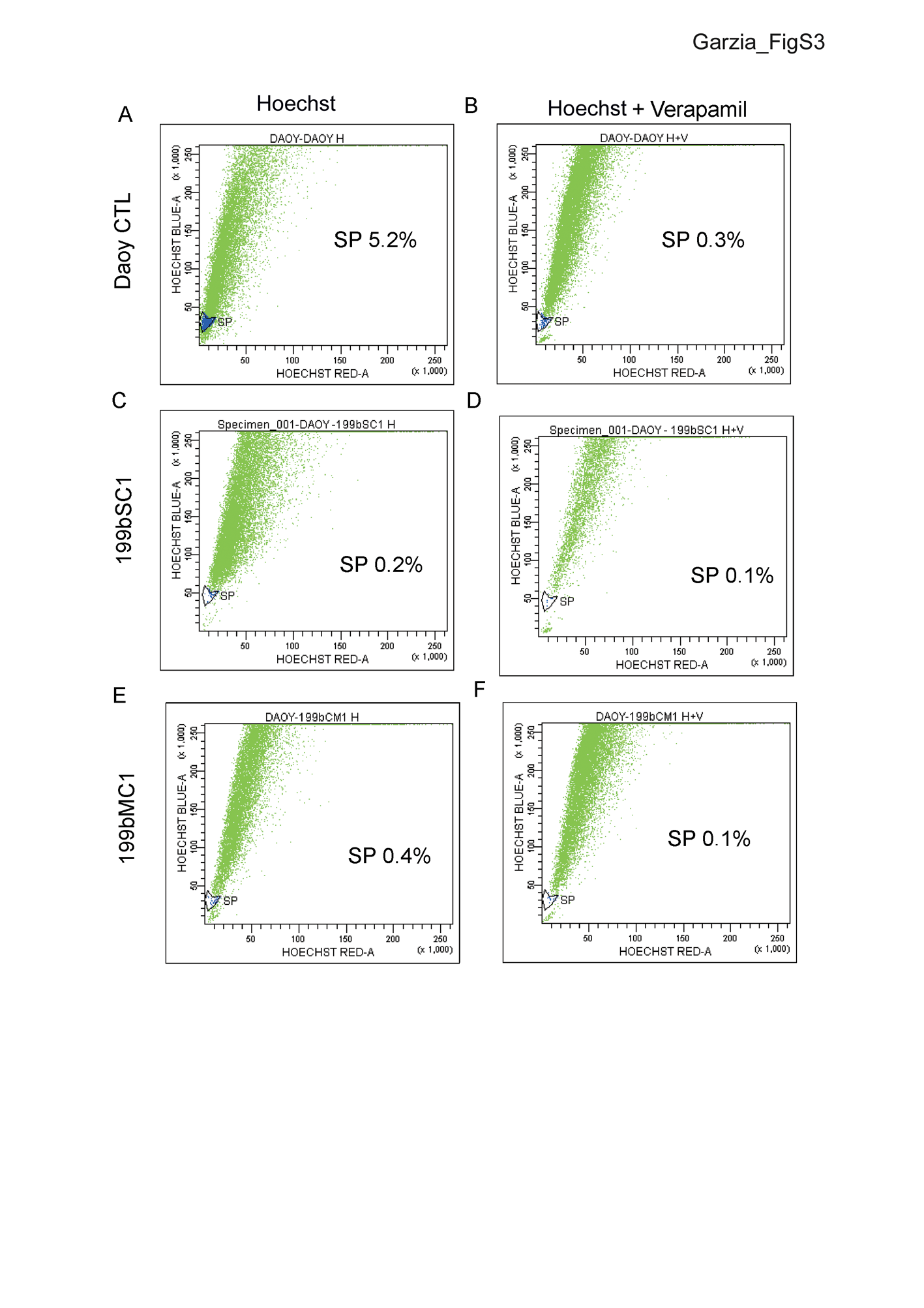

Supplement: Figure S3 — FACS analysis for the role of miR-199b-5p on the Daoy cell side population. A, C, E) Decrease in the SP cells of the Daoy 199bSC1 stable clone, as determined by Hoechst 33342 staining. B, D, F) Addition of verapamil to force dye incorporation also in the SP cells. The Daoy cells showed a 5.2% fraction of SP cells, while the stable 199bSC1 and 199bMC1 clones over-expressing miR-199b-5p do not show significant levels of SP cells (0.2%, 0.4%, respectively) (8.54 MB TIF) [file pone.0004998.s004.tif]

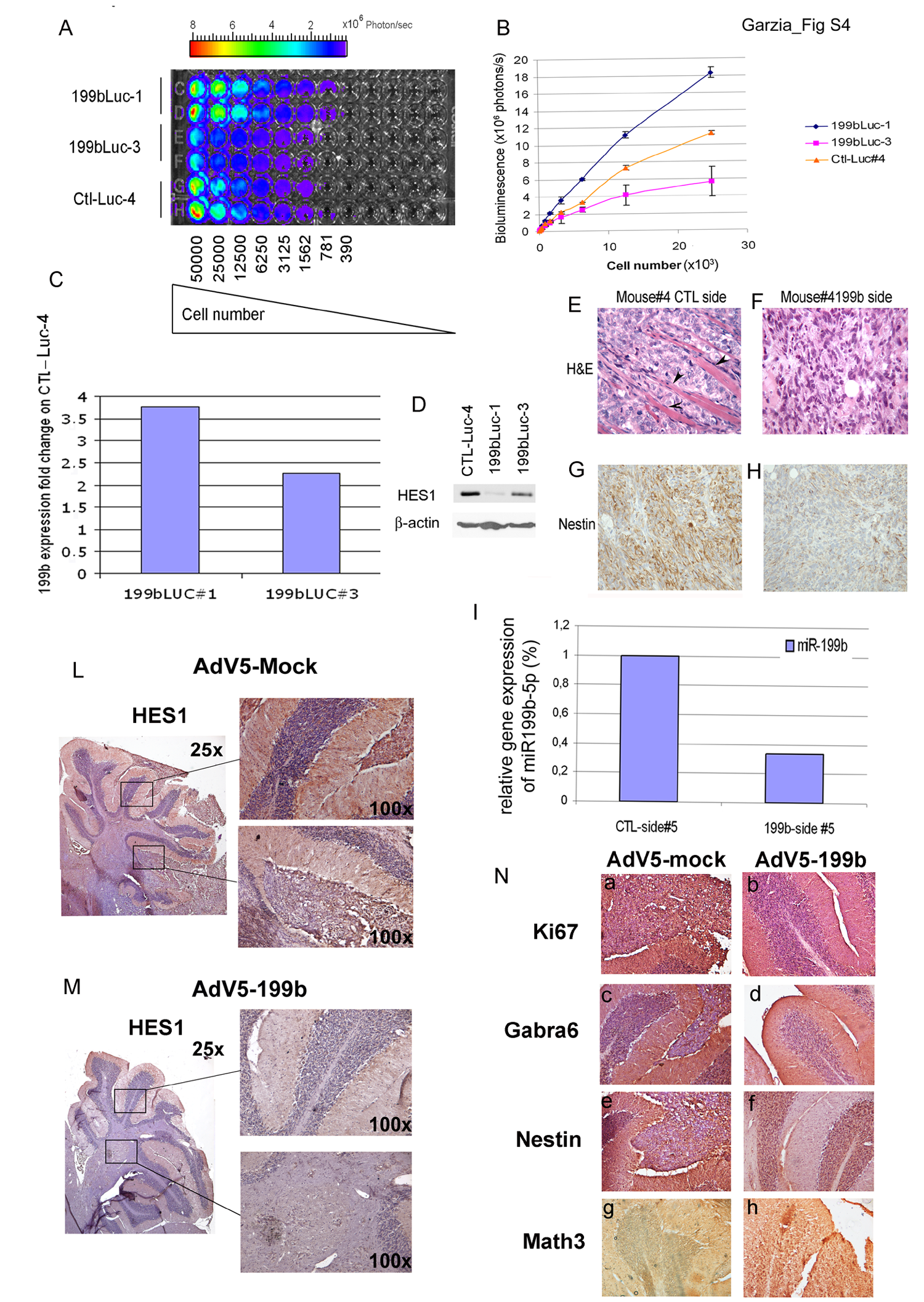

Supplement: Figure S4 — Creation of bioluminescent Daoy cells over-expressing miR-199b-5p for in-vivo studies. The Daoy stable clone with the empty vector (Ctl) and the Daoy stable clone over-expressing miR-199b-5p (199b) were transfected with a luciferase expression vector (Luc), generating, respectively, the Ctl-Luc#4 clone, and the199bLuc-1 and 199b-Luc-3 clones. These were all evaluated for their expression of bioluminescence when incubated with the enzyme substrate, luciferin. A) Light emission of serial dilutions of the cells indicated in a microplate. The bioluminescence signal was acquired by an IVIS 200 Imaging System (Xenogen Corp. Alameda, CA). B) Correlation between cell number and photon emission. The 199b-Luc1 and Ctl-Luc#4 clones where used for the in-vivo studies, with the data shown as means±SD from two independent experiments C) Representative data of the expression of miR-199b-5p in the luciferase clones, relative to the luciferase clone carrying the empty vector. D) Representative immunoblot analysis showing lowered expression of HES1 in the 199b-Luc clones, compared to the empty-vector clone. E, F) Hematoxylin and eosin staining of xenografts derived from mouse #4: the control-injected side shows the xenograft infiltrating the muscle tissue, while the 199b-injected side does not. G, H) Nestin is a marker of neuroblasts, and its expression correlates with a lesser differentiated state: the xenograft from the mouse #4 199b-injected side shows a decrease in Nestin positivity. Nestin staining was perfomed by immunohistochemistry with a polyclonal antibody (Abcam). Magnification, 100×. I) Representative gene expression levels of the transgene miR-199b-5p in the tumor explant from mouse #5. The over-expression of miR-199b-5p was lost. L, M, N) Immunohistochemistry with anti-HES1, anti-KI67, anti-Gabra6, anti-Nestin, anti-Math3 antibodies and hematoxylin staining of the xenografts from the cerebellum of AdV5-Mock and AdV5-199b mice. Significant expression of Hes1, Ki67, Nesti [file pone.0004998.s005.tif]

A

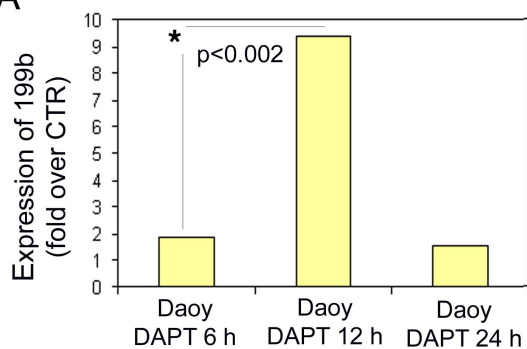

B

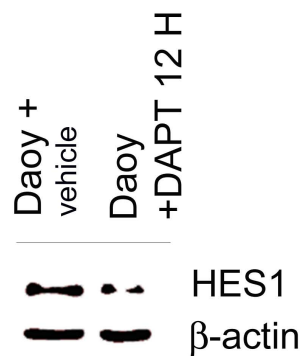

C

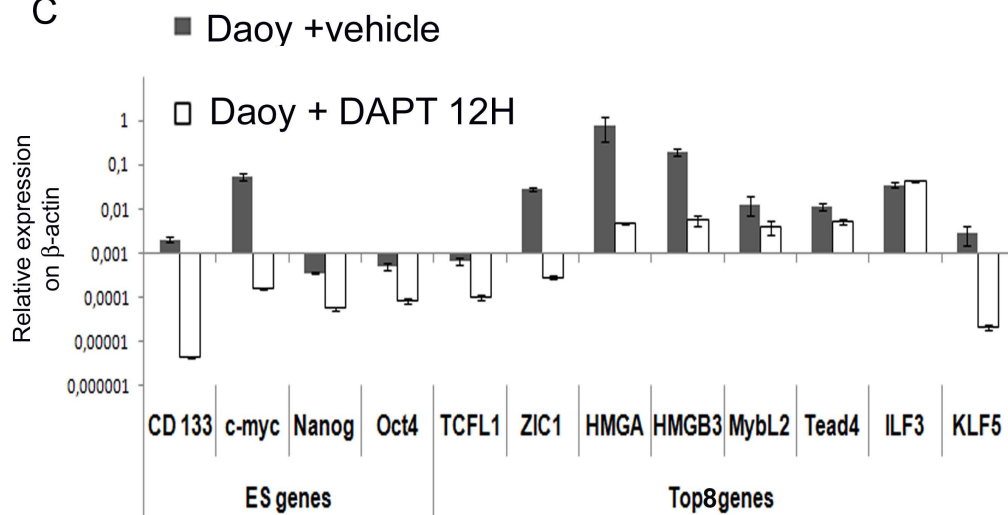

D

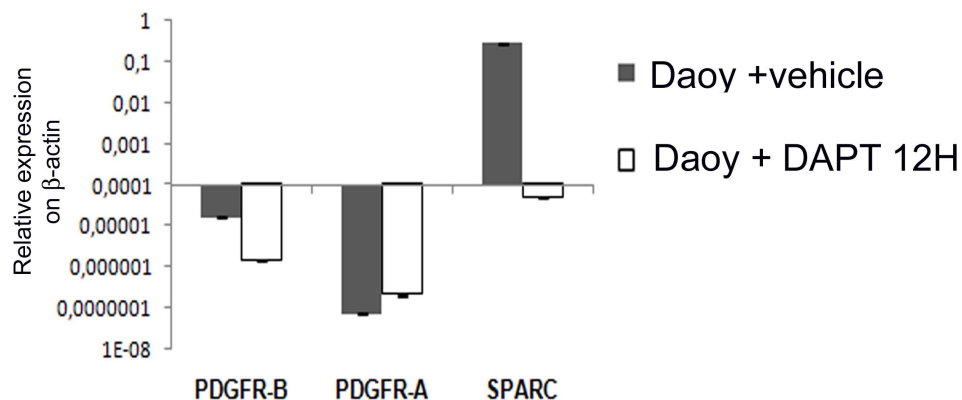

Supplement: Figure S5 — Over-expression of endogenous miR199b upon DAPT treatment induces down-regulation of genes involved to embryonic stem cells and cancer stem cells in the Daoy cell line. A) Representative data of a time course experiment of DAPT treatment of Daoy cell lines (6 h, 12 h, 24 h) with media supplemented and replaced every 4 h, and fold of expression of miR199b determined using quantitive real time detection, relative to time 0. B) Representative Western blot showing HES1 down-regulation after 12 h of induction of DAPT in the Daoy cell line. C) Relative gene expression levels of CD133, c-Myc Oct4, KFL5, Nanog, TCF7L1, HMGA1, HMGB3, ZIC1, MYBL2, TEAD4, ILF3 in the Daoy cell line with and without DAPT treatment for 12 h. D) Relative gene expression of PDGFR-A, PDGFR-B and SPARC in the Daoy cell line with and without DAPT treatment for 12 h The primers used and their DDct values of relative expression using real-time cDNA quantitative detection, are listed in Supporting Table S3. (0.47 MB PDF) [file pone.0004998.s006.pdf]

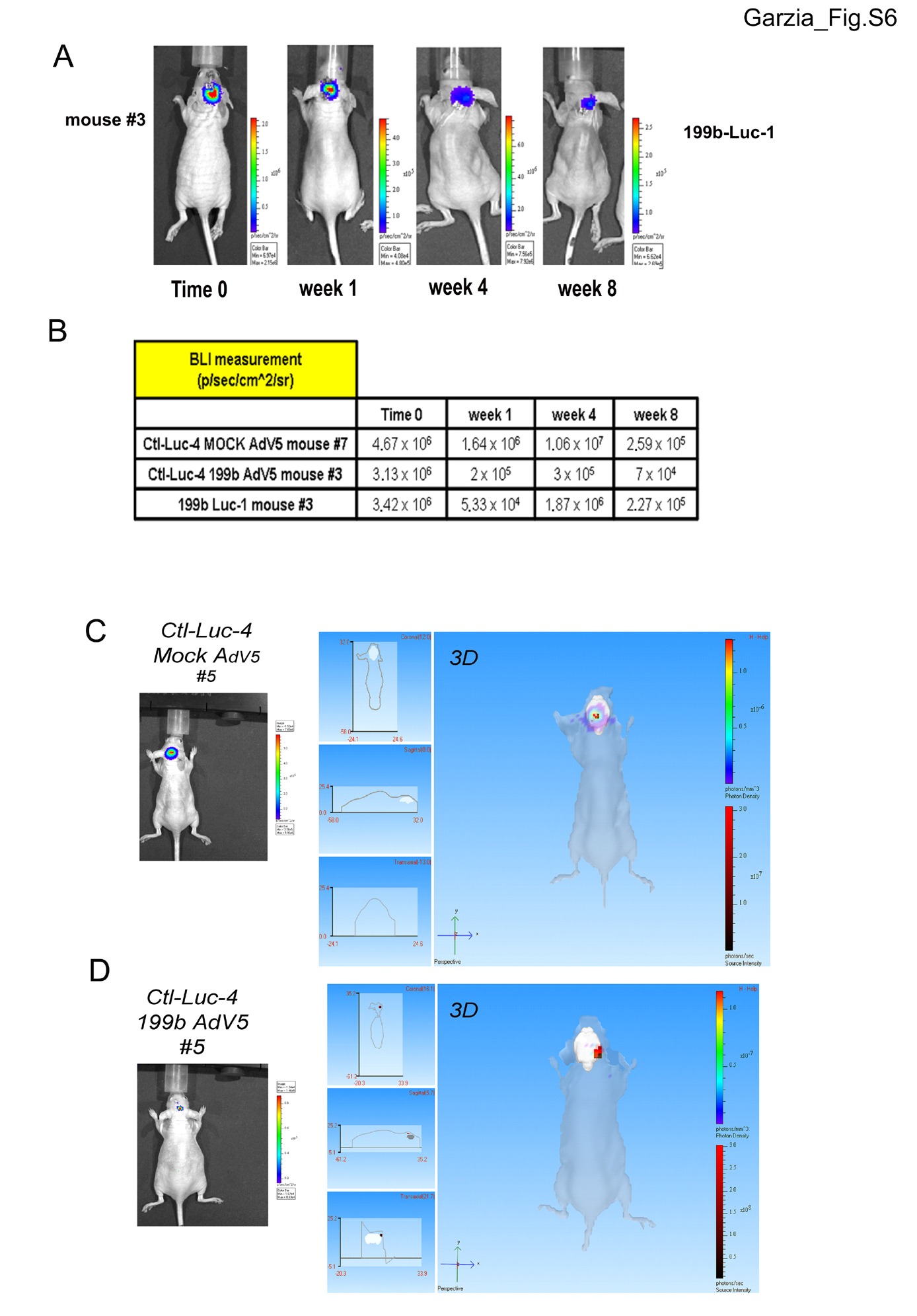

Supplement: Figure S6 — Mir-199-b-5p interferes with the engraftment potential of Daoy cells injected into mouse cerebellum. A) In-vivo bioluminescence analyses using IVIS 3D of 199b-Luc1 mouse xenografted with Daoy Luc1 cell line overexpressing miR-199b by stable clone analyses. (see mouse #3; see additional data in Fig. 4F, main text). The mouse was scanned once a week for a total of 8 weeks. B) BLI measurements (photon/sec/cm-2) from mice carrying cells infected with ADV5-199b show reductions to week 8. C) Images taken by IVIS Spectrum at 560 nm and 660 nm to generate the topography of the subject. Clt-Luc-4 AdV5-Mock #5 xenografted mice shown on a 3D axis (x,y,z), with the extension of the tumor burden shown in comparison with a digital mouse atlas that enabled the display of the 3D skeleton and organs on the 3D reconstruction, using Living Images software. (See also Movie S1 and Figure 4 panel F for BLI fold changes values) D) As described above, analyses are taken on Ctl-luc AdV5 199b #5 xenografted mice (See also Movie S2 and Figure 4 panel F for BLI fold changes values). (8.30 MB TIF) [file pone.0004998.s007.tif]
