# Supplementary material for: MicroRNA-199b-5p Impairs Cancer Stem Cells through Negative Regulation of HES1 in Medulloblastoma
Source: PLoS One. 2009 Mar 24;4(3):e4998. doi: 10.1371/journal.pone.0004998 (PMC2656623; doi:10.1371/journal.pone.0004998)
Supplement: Table S1 — Cancer related targets of miR-199b-5p. MiRanda and Pita algorithms were applied to the selected “cancer-related” miR-199b-5p targets. The gene targets predicted by both of these algorithms are listed, including: MIRANDA score, P values, Pita analyses with sequence matches and Delta-Delta G values. (0.04 MB DOC) [file pone.0004998.s008.doc]

|  | **Table 1S | Cancer-related Targets list of human miR-199b-5P** | | | | | |
| --- | --- | --- | --- | --- | --- | --- |
|  | **Gene Target** | **Gene Function** | **Miranda Analysis** | | **Pita Analysis** | |
|  |  |  | Score | P-value | Seed | ΔΔG |
|  | HES1 | Transcription factor | 18,2355 | 5,80E-03 | 8:0:1 | -8,92 |
|  | NTRK1 | Neurotrophic tyrosine kinase receptor | 19,4586 | 1,70E-03 | 8:0:1 | 1,21 |
|  |  |  |  |  |  |  |
|  | CCNL1 | Clyclin L1 | 17,4364 | 3,60E-05 | 8:0:0 | -10.28 |
|  | CDK9 | Cyclin- dependent kinase 9 | 16,488 | 1,40E-04 | 8:0:0 | -5.03 |
|  | HIRA | HIR histone cell cycle regulation defective homolog A | 16,224 | 2,10E-04 | 8:0:1 | -2.52 |
|  | HOXC5 | Homeobox protein Hox-C5 | 16,157 | 8,21E-03 | 8:0:1 | 0,09 |
|  | MAP2K5 | Dual specificity mitogen-activated protein kinase kinase 5 | 16,2892 | 4,35E-02 | 8:0:1 | -4.13 |
|  | MAP3K12 | Mitogen-activated protein kinase kinase kinase 12 | 15,8078 | 6,10E-03 | 8:0:1 | -6.37 |
|  | NANOG | Homeobox protein NANOG | 17,3329 | 0,01486 | 8:0:1 | -7.65 |
|  | NHLH2 | Helix-loop-helix protein 2 (HEN2) | 16,0155 | 4,62E-03 | 8:0:0 | -7.59 |
|  | OTX1 | Homeobox protein OTX1 | 16,1851 | 2,04E-04 | 8:0:1 | -8.68 |
|  | GSK3B | Glycogen synthase kinase-3 beta | 16,4041 | 3,80E-02 | 8:0:0 | 0,03 |
|  |  |  |  |  |  |  |
