# Supplementary material for: MicroRNA-199b-5p Impairs Cancer Stem Cells through Negative Regulation of HES1 in Medulloblastoma
Source: PLoS One. 2009 Mar 24;4(3):e4998. doi: 10.1371/journal.pone.0004998 (PMC2656623; doi:10.1371/journal.pone.0004998)
Supplement: Table S2 — Patients characteristics recruited for the study. A) In columns Age at diagnosis in month, Follow-up time in months, State at last news, Mestatasis (M) stage, Histology, Relative expression of miR-199b-5p compared to the level of (U6) and 2−Delta Ct values. B) Normal healthy cerebellum mRNA from the Brain and tissue Bank, University of Maryland, Baltimore (USA) were used for this study. In columns, ID of material, age in year and relative expression of miR-199b-5p compared to the level of (U6) and 2−Delta Ct values. (0.15 MB DOC) [file pone.0004998.s009.doc]

|  | |  |  | |  | |  | |  |  |
| --- | --- | --- | --- | --- | --- | --- | --- | --- | --- | --- |
| **Table S2 A** | |  |  | |  | |  | |  |  |
| **Patient** | | **Age at diagnosis (months)** | **Follow-up (months)** | | **State at last news** | | **M stage (Chang)** | | **Histology** | **HSA-mir-199b-5p relative expression (U6) 2^-ΔCt** |
| MB102 | | 108 | 32.8 | | dead from disease | | M3 | | classic | 0.00099561 |
| MB108 | | 96 | 19.5 | | CR1 | | M0 | | desmoplastic | 0.09001227 |
| MB106 | | 50 | 4 | | dead from disease | | M0 | | classic | 0.010613742 |
| MB111 | | 9 | 72.2 | | CR1 | | M0 | | desmoplastic | 0.120138583 |
| MB114 | | 21 | 74.6 | | CR1 | | M0 | | desmoplastic | 0.000383771 |
| MB119 | | 93 | 32.8 | | CR1 | | M1 | | classic | 0.003199194 |
| MB120 | | 108 | 27.5 | | CR1 | | M0 | | classic | 0.218701701 |
| MB123 | | 37 | 13.6 | | dead from disease | | M3 | | classic | 0.006959284 |
| MB126 | | 13 | 74.8 | | CR2 | | M0 | | post treatment | 0.067296233 |
| MB128 | | 78 | 31.7 | | CR1 | | M0 | | desmoplastic | 0.076875702 |
| MB133 | | 158 | 87 | | CR1 | | M0 | | classic | 0.025786457 |
| MB137 | | 93 | 84 | | CR1 | | M0 | | classic | 0.019919437 |
| MB79 | | 36 | 14.1 | | dead from disease | | M0 | | medullo-myoblastoma | 0.194622694 |
| MB80 | | 96 | 43.1 | | CR1 | | M2 | | desmoplastic | 0.000152879 |
| MB81a | | 109 | 14 | | dead from disease | | M0 | | large cell | 0.019211033 |
| MB95 | | 41 | 32.1 | | CR1 | | M0 | | classic | 0.002433079 |
| MB96 | | 44 | 42 | | dead from disease | | M0 | | classic | 0.004037267 |
| MB99 | | 99 | 21.9 | | dead from disease | | M0 | | post treatment | 0.015258058 |
| N1 | | 69 | 49 | | CR1 | | M2 | | classic | 0.005729706 |
| N2 | | 90 | 20 | | CR1 | | M0 | | classic | 0.206256346 |
| N3 | | 26 | 34 | | CR1 | | M0 | | desmoplastic | 1.770829272 |
| N4 | | 124 | 14 | | CR1 | | M0 | | classic | 0.014508728 |
| N5 | | 64 | 36 | | PR | | M0 | | classic | 0.386116248 |
| N6 | | 35 | 1 | | dead from disease | | M3 | | large cell | 0.007290413 |
| N7 | | 76 | 22 | | CR1 | | M0 | | classic | 0.009490309 |
| N8 | | 169 | 26 | | CR1 | | M0 | | classic | 0.066566636 |
| N9 | | 128 | 10 | | CR1 | | M0 | | classic | 0.379446328 |
| N10 | | 29 | 20 | | PD | | M0 | | classic | 0.032431549 |
| N11 | | 140 | 34 | | CR1 | | M0 | | classic | 0.097916916 |
| N12 | | 34 | 19 | | PD | | M0 | | desmoplastic | 0.034231549 |
| MDT-MB-1 | | 25 | 6 | | dead from disease | | M+(1-2-3) | |  | 1.574119241 |
| MDT-MB-2 | |  |  | |  | | M+(1-2-3) | |  | 0.012369684 |
| MDT-MB-3 | |  |  | |  | | M0 | |  | 0.408535751 |
| MDT-MB-4 | |  |  | |  | | M0 | |  | 0.014345797 |
| MDT-MB-5 | |  |  | |  | | M+(1-2-3) | |  | 0.117424185 |
| MDT-MB-6 | | 27 | 33 | | dead from disease | | M0 | | desmoplastic | 1.924140125 |
| MDT-MB-7 | | 88 | 14 | | dead from disease | | M0 | | anaplastic | 0.369604567 |
| MDT-MB-8 | | 124 | 39 | | alive | | M0 | | anaplastic | 0.499210685 |
| MDT-MB-9 | | 39 | 95 | | alive | | M0 | |  | 0.085790892 |
| MDT-MB-38 | | 60 | 78 | | alive | | M0 | |  | 0.019155739 |
| MDT-MB-46 | |  |  | |  | | M0 | |  | 1.875853287 |
| MDT-MB-50 | |  |  | |  | | M+(1-2-3) | |  | 0.043612817 |
| MDT-MB-71 | |  |  | |  | | M0 | |  | 0.07458206 |
| MDT-MB-100 | | 32 | 34 | | alive | | M0 | |  | 0.645850534 |
| MDT-MB-102 | | 26 | 33 | | alive | | M+(1-2-3) | | anaplastic | 0.020193158 |
| MDT-MB-103 | | 65 | 33 | | alive | | M0 | |  | 0.130267521 |
| MDT-MB-118 | | 109 | 32 | | alive | | M+(1-2-3) | | anaplastic | 1.776616235 |
| MDT-MB-124 | |  |  | |  | | M0 | |  | 0.902823686 |
| MDT-MB-131 | |  |  | |  | | M+(1-2-3) | |  | 0.277894072 |
| MDT-MB-154 | |  |  | |  | | M+(1-2-3) | |  | 0.03848591 |
| MDT-MB-174 | |  |  | |  | | M+(1-2-3) | |  | 0.016130305 |
| MDT-MB-175 | |  |  | |  | | M0 | |  | 0.821566771 |
| MDT-MB-177 | |  |  | |  | | M+(1-2-3) | |  | 0.004737906 |
| MDT-MB-193 | |  |  | |  | | M+(1-2-3) | |  | 0.142617576 |
| MDT-MB-194 | |  |  | |  | | M+(1-2-3) | |  | 0.484220261 |
| MDT-MB-201 | |  |  | |  | | M0 | |  | 0.053878318 |
| MDT-MB-207 | | 61 | 110 | | alive | | M0 | |  | 0.025498266 |
| MDT-MB-220 | | 69 | 20 | | alive | | M0 | |  | 1.119673662 |
| MDT-MB-222 | | 72 | 20 | | alive | | M+(1-2-3) | |  | 0.050742316 |
| MDT-MB-223 | | 131 | 20 | | alive | | M0 | |  | 0.374030612 |
| MDT-MB-224 | | 167 | 13 | | dead from disease | | M0 | | anaplastic large cell | 1.02682E-10 |
|  | |  |  | |  | |  | |  |  |
|  | **Tab. S2 B** | | | | | | |  | | |
|  | **Patient code** | | | **Age (years)** | | **HSA-mir-199-5p relative expression (U6) 2^-ΔCt** | |  | | |
|  | **1 year old** | | |  | |  | |  | | |
|  | 1210 | | | 0.2 | | 0.028323341 | |  | | |
|  | 1157 | | | 0.02 | | 0.091384778 | |  | | |
|  | 1102 | | | 0.39 | | 0.010811309 | |  | | |
|  | 779 | | | 0.005 | | 0.009558291 | |  | | |
|  | 814 | | | 1.41 | | 0.031308608 | |  | | |
|  | **13-16 years old** | | | | |  | |  | | |
|  | 1297 | | | 15.81 | | 0.001938772 | |  | | |
|  | 1158 | | | 16.63 | | 0.001053237 | |  | | |
|  | 1065 | | | 15.29 | | 0.002201934 | |  | | |
|  | 1024 | | | 14.6 | | 0.000768088 | |  | | |
|  | 931 | | | 13.12 | | 0.003939267 | |  | | |
|  | 142 | | | 16.73 | | 0.000206487 | |  | | |
|  |  | | |  | |  | |  | | |
