# Supplementary material for: MicroRNA-199b-5p Impairs Cancer Stem Cells through Negative Regulation of HES1 in Medulloblastoma
Source: PLoS One. 2009 Mar 24;4(3):e4998. doi: 10.1371/journal.pone.0004998 (PMC2656623; doi:10.1371/journal.pone.0004998)
Supplement: Table S3 — Stem and cancer stem genes, and genes associated with MB tumor development in Daoy cell lines over-expressing endogenous miR-199b-5p under treatment with DAPT. The genes involved in stem cell biology selected for the study of their expression after over-expression of miR-199b-5p. The Unigene ID and sequence of primers used for real-time PCR are shown, the 2−Delta Ct values are obtained from the analysis of Daoy cells treated with DAPT for 12 h and analysed with the 7700 Real Time TaqMan Applied Biosystem. (0.05 MB DOC) [file pone.0004998.s010.doc]

| **Table S3** |  |  |  |  |  |
| --- | --- | --- | --- | --- | --- |
| **Gene name** | **Unigene ID** | **Primer Forward** | **Primer Reverse** | **Daoy + vehicle** | **Daoy + DAPT 12h** |
| c-Myc | Hs.202453 | ATGAGGAGACACCGCCCAC | AACATCGATTTCTTCCTCATCTTCTT | 0.0987097 | 0.0542671 |
| Nanog | Hs.661360 | GCAAATGTCTTCTGCTGAGATGC | GCTGTCCTGAATAAGCAGATCCAT | 0.0001832 | 0.0004488 |
| CD 133 | Hs.614734 | CTATGTGGTACAGCCGCGTG | TAATCAATTTTGGATTCATATGCCTTC | 0.0020653 | 0.0012368 |
| Oct4 | Hs.249184 | ACTGCAGCAGATCAGCCACA | TGGCGCCGGTTACAGAAC | 0.0001200 | 0.0007941 |
| PDGFR-B | Hs.509067 | AGGTTGCTGACGAGGGCC | GGTGTTGACTTCATTCAGGGTG | 0.0006059 | 0.0000606 |
| PDGFR-A | Hs.74615 | TCAAGGCAGAAATAGGCAGCA | TGGACGTCGATCAGGTCCA | 0.0000003 | 0.0000015 |
| SPARC | Hs.708558 | TTGCCTGGACTCTGAGCTGA | GGGTGACCAGGACGTTCTTG | 0.3431687 | 0.0797725 |
| ILF3 | Hs.465885 | CCGACACGCCAAGTGGTT | ACACAAGACTTCAGCCCGTTG | 0.0352218 | 0.0425171 |
| MYBL2 | Hs.179718 | AGCAAGTGCAAGGTCAAATGG | GGCCCTCAGCTGCTCGT | 0.0130289 | 0.0039321 |
| TEAD4 | Hs.94865 | TCGGACGAGGAGGGCAAGATG | GATGTAGCGGGCAATCAGCT | 0.0117936 | 0.0051764 |
| TCF7L1 | Hs.516297 | CCGCGGGACTATTTCGC | AAAGAACGCGCTGTCCTGAG | 0.0006742 | 0.0001026 |
| HMGA1 | Hs.518805 | AAAAACAAGGGTGCTGCCAA | CCTTCCTGGAGTTGTGGTGGT | 0.7977002 | 0.0048793 |
| ZIC1 | Hs.598590 | CAGTTCGCTGCGCAAACA | GAGCCCTGCGAGGAGGAT | 0.0283592 | 0.0002784 |
| HMGB3 | Hs.19114 | TTTTCCAAGAAGTGCTCTGAGAGG | TTTCTCTTTCCCGGACATCG | 0.1946868 | 0.0055773 |
| KLF5 | Hs.508234 | GCATCCACTACTGCGATTACCC | TGAGAAGACTTGGTATAAACTTTTGTGC | 0.0027895 | 0.0236774 |
|  |  |  |  |  |  |
